# Multiple binding modes underlie *Cannabis sativa* cannabinoids recognition by peroxisome proliferator-activated receptor gamma

**DOI:** 10.3389/fbinf.2026.1893303

**Published:** 2026-08-10

**Authors:** N. R. Carina Alves, Justina Miranda, Lautaro D. Alvarez

**Affiliations:** 1 Departamento de Química Biológica, Facultad de Ciencias Exactas y Naturales, Universidad de Buenos Aires, Ciudad Universitaria, Buenos Aires, Argentina; 2 UMYMFOR, CONICET - Universidad de Buenos Aires, Ciudad Universitaria, Buenos Aires, Argentina

**Keywords:** binding modes, cannabinoids, docking, molecular dynamics simulation, PPARγ

## Abstract

**Introduction:**

Peroxisome proliferator-activated receptor gamma (PPARγ) is a ligand-activated nuclear receptor with broad therapeutic relevance across various pathologies, including type 2 diabetes, obesity, cancer, and inflammatory disorders. Cannabinoids are a class of terpene-phenolic compounds from *Cannabis sativa* L. that have been shown to act as partial agonists of PPARγ. Among them, the acidic forms Δ^9^-tetrahydrocannabinolic acid (THCA) and cannabidiolic acid (CBDA) display higher potency than their decarboxylated counterp arts Δ^9^-tetrahydrocannabinol (THC) and cannabidiol (CBD). Despite experimental evidence supporting direct PPARγ-cannabinoid interaction, the molecular determinants governing ligand recognition within the binding pocket have not yet been comprehensively investigated.

**Methods:**

A combination of molecular docking and molecular dynamics simulations was employed to characterize the binding modes of THC, CBD, THCA, and CBDA within the PPARγ ligand-binding domain. Docking calculations were performed on a curated set of 70 PPARγ crystal structures co-crystallized with structurally diverse ligands, exploiting thus the conformational variability of the binding pocket. The best-ranked solutions were subjected to 500 ns MD simulations and evaluated on the basis of ligand stability, persistence of polar and aromatic-aromatic interactions with the receptor, and energetic contributions estimated by MM/GBSA. Three candidate binding modes per ligand were selected and their trajectories extended to 1,000 ns.

**Results:**

All four cannabinoids yielded at least one stable binding mode at the microsecond timescale. The cannabinoids THCA and CBDA displayed a greater number of stable binding modes than THC and CBD, a result consistent with the higher potency previously reported for these compounds in experimental studies. This behavior may be attributable to the formation of salt bridges with basic residues in the binding pocket.

**Conclusion:**

Our findings provide a structural framework for understanding cannabinoid recognition by PPARγ. The ability of these compounds to adopt multiple binding modes may contribute to their partial agonist profile, opening new avenues for the rational design of selective PPARγ modulators with improved therapeutic properties.

## 1 Introduction

Peroxisome proliferator-activated receptors (PPARs) are ligand-activated nuclear receptors (NRs) that function as transcriptional regulators of key metabolic and inflammatory pathways, including glucose and lipid homeostasis, adipogenesis, and immune responses (Hernandez-Quiles et al., 2021; Vázquez-Carrera and Wahli, 2022; Wagner and Wagner, 2023). Three PPAR isoforms have been identified: PPARα, PPARβ/δ, and PPARγ, each characterized by distinct tissue distribution patterns and physiological functions. Among them, PPARγ is of particular interest due to its significant therapeutic relevance as a molecular target for the treatment of diverse entities, including type 2 diabetes, obesity, cancer, and inflammatory disorders (Chi et al., 2021; Frkic et al., 2021; Stark et al., 2021; Kounatidis et al., 2025). Structurally, the inherent flexibility and adaptability of PPARγ ligand-binding pocket (LBP) enable the accommodation of a broad range of endogenous ligands, such as fatty acids and prostaglandin derivatives, which typically exhibit low binding affinity and preferentially act as partial rather than full agonists.

Although the development of synthetic ligands capable of modulating PPARγ activity began decades ago, leading to the generation of potent agonists such as thiazolidinediones (e.g., rosiglitazone and pioglitazone), the development of therapeutic agent devoid of serious adverse effects remains elusive. Accumulating evidence indicates that full PPARγ agonists disrupt metabolic regulatory pathways, resulting in adverse outcomes such as weight gain, fluid retention, edema, and an increased risk of heart failure (Wallach et al., 2020; Giglio et al., 2022).

**FIGURE 1:**
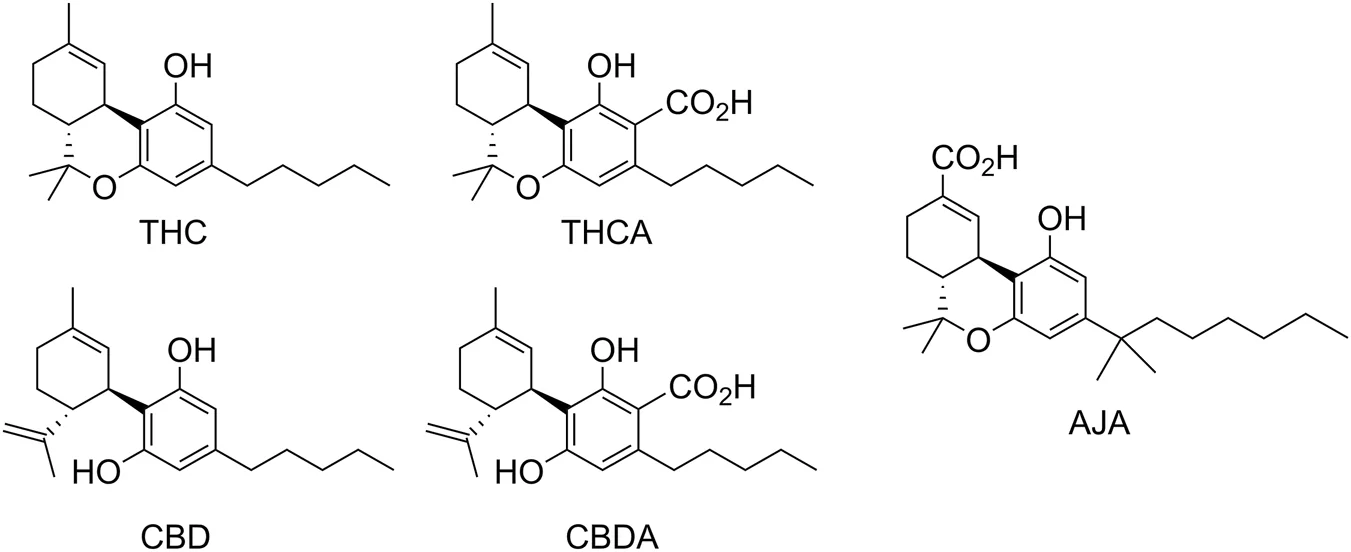
Natural cannabinoids THC, CBD, THCA, and CBDA and synthetic analog ajulemic acid, AJA.

Cannabinoids constitute a class of compounds built around a terpene phenolic scaffold, which is characteristic of *Cannabis sativa* L. Over 120 different cannabinoids have been isolated and characterized (Hanuš et al., 2016), many of which are capable of modulating the activity of a wide variety of human proteins (Blebea et al., 2024; Wright, 2024). The most abundant cannabinoids are Δ^9^-tetrahydrocannabinol (Δ^9^-THC, hereafter THC) and cannabidiol (CBD), which arise from the decarboxylation of their corresponding acidic precursors present in the plant matrix, namely, Δ^9^-tetrahydrocannabinolic acid (THCA) and cannabidiolic acid (CBDA), respectively. The chemical structures of these four cannabinoids are shown in Figure 1. The most extensively studied and well-established mechanism of cannabinoid action involves their interaction with the cannabinoid receptors CB1 and CB2, members of the G protein-coupled receptor (GPCR) family (Shahbazi et al., 2020). However, recent studies have demonstrated that cannabinoids can also function as ligands for a variety of other receptor families, particularly the PPARs. Indeed, binding assays, reporter gene expression studies, endogenous gene regulation analysis, and *in vivo* experiments have shown that THC, CBD, THCA, and CBDA modulate PPARγ activity (O’Sullivan et al., 2009; Carroll et al., 2012; Vara et al., 2013; Scuderi et al., 2014; Takeda et al., 2014; Nadal et al., 2017; D’Aniello et al., 2019; Palomares et al., 2020; Chang et al., 2022; Hirao-Suzuki et al., 2022), acting as partial agonists of this receptor. Furthermore, the direct involvement of PPARγ was confirmed through the use of selective antagonists, which abolished the effect elicited by these cannabinoids (Scuderi et al., 2014; Chang et al., 2022). Notably, acidic cannabinoids exhibit higher binding affinities and potencies than their decarboxylated counterparts, suggesting that the carboxyl group plays an active role in ligand recognition within the PPARγ binding pocket (Nadal et al., 2017). Nevertheless, even though some previous studies have addressed the binding mode of THCA and CBDA (D’Aniello et al., 2019; Palomares et al., 2020), the precise molecular determinants governing the interaction between the four principal cannabinoids and PPARγ remain to be established.

PPARs display the canonical NR architecture, consisting of an N-terminal domain with transcriptional activity (AF-1), a central DNA-binding domain (DBD) containing zinc finger motifs responsible for recognition of PPAR response elements (PPREs), and a C-terminal ligand-binding domain (LBD) (Chandra et al., 2008). The LBD comprises a predominantly α-helical fold and harbors both the activation function 2 (AF-2) motif, which mediates ligand-dependent co-regulator recruitment, and the interface required for heterodimerization with retinoid X receptor (RXR). Structurally, the ligand-binding pocket (LBP) of PPARγ forms a large, Y-shaped and conformationally adaptable cavity, whose volume and flexibility enable the accommodation of molecules with diverse structural and chemical features. Consistently, the Protein Data Bank (PDB) currently reports more than 250 crystallographic structures of PPARγ in complex with structurally diverse ligands, underscoring the pronounced plasticity of the LBP and its ability to recognize molecules of varying sizes and functional groups.

As in most NRs, the molecular mechanism underlying PPAR function is primarily explained by ligand-induced modulation of the conformational dynamics of the C-terminal helix 12 (H12). Together with helices H3 and H4, H12 defines the AF-2 superficial cavity, which constitutes the primary interaction platform for co-regulator recruitment. Numerous theoretical and experimental approaches have shown that, in PPARγ, H12 populates a dynamic ensemble of conformations, spanning from “open” states that favor co-repressor association to “closed” states in which the AF-2 motif surface is optimally configured for coactivator recruitment (Kroker and Bruning, 2015; Chrisman et al., 2018; Frkic et al., 2018; Falbo et al., 2025). Intermediate conformations have also been identified and are associated with distinct co-regulator binding profiles. At the molecular level, differences in ligand-receptor interactions, including hydrogen-bonding networks and hydrophobic or aromatic contacts, modulate the conformational landscape sampled by H12 and thereby determine the transcriptional activity profile of the receptor.

From a pharmacological perspective, the development of safer PPARγ-targeting compounds aims to avoid rigid stabilization of H12 in its fully closed conformation, a state associated with extensive coactivator recruitment and full transcriptional activation, both of which have been linked to adverse metabolic effects. Accordingly, current strategies focus on the development of selective PPARγ modulators (SPPARγMs) capable of reshaping the dynamic equilibrium of H12 by stabilizing alternative conformational subpopulations that preserve beneficial activity while attenuating excessive coactivator recruitment (Bruning et al., 2007; Zhang et al., 2007; Kroker and Bruning, 2015). In this context, the partial agonist behavior of cannabinoids, together with their well-documented therapeutic safety profile, positions these compounds as promising candidates for PPARγ modulation aimed at dissociating therapeutic efficacy from undesirable adverse effects.

In this work, to investigate the molecular determinants underlying the interaction between PPARγ and cannabinoids, a combination of docking and molecular dynamics (MD) simulations was employed to evaluate the binding modes of THC, CBD, THCA, and CBDA. Specifically, the study focused on characterizing the predominant binding orientations adopted by these cannabinoids within the LBP, as well as the ligand-receptor interactions governing complex stability. Through this approach, this work aimed to advance the understanding of the structural basis of the PPARγ-cannabinoid interactions and to provide insight into the potential of these natural compounds as modulators of PPARγ activity.

## 2 Computational methods

### 2.1 Overview of the computational workflow

**FIGURE 2:**
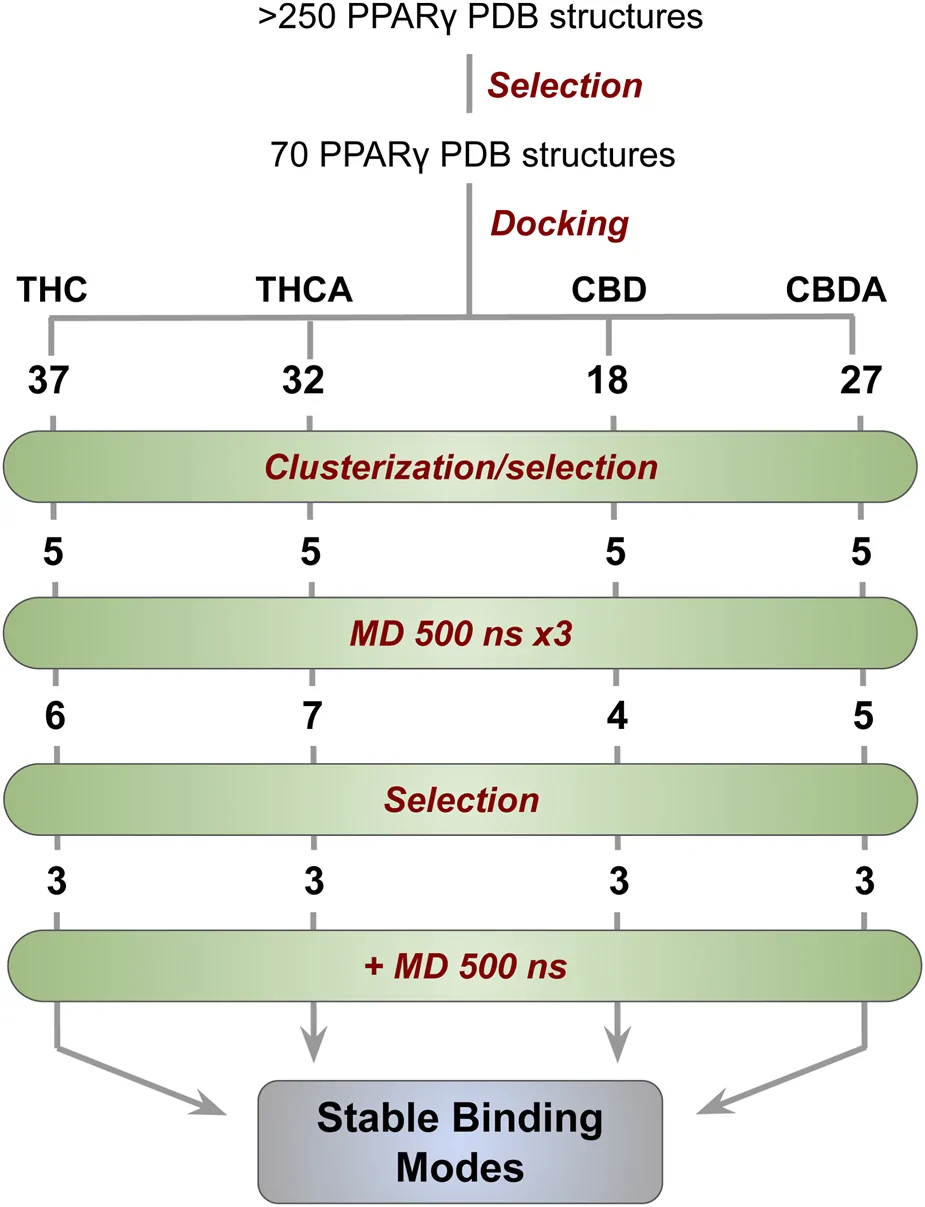
Workflow for the identification of stable binding modes of natural cannabinoids in the PPARγ LBD. The number of solutions or binding modes at each stage is indicated for each ligand.

Figure 2 outlines the workflow employed to identify putative cannabinoid binding modes within the PPARγ LBD. Briefly, the strategy consisted of selecting a representative set of crystal receptor structures, generating initial complexes through docking, and progressively filtering them based on the stability and ligand-receptor interactions observed during MD simulations. Thus, starting from a curated selection of 70 PPARγ crystal structures from over 250 PDB entries (www.rcsb.org), docking calculations yielded 37, 32, 18, and 27 acceptable solutions for THC, THCA, CBD, and CBDA, respectively. Following RMSD-based clusterization and pose selection, five representative complexes per ligand were subjected to 500 ns MD simulations carried out in three independent replicates, leading to the characterization of 6, 7, 4, and 5 binding modes for THC, THCA, CBD, and CBDA, respectively. Subsequently, three modes per ligand were then selected based on ligand stability, persistence of ligand-receptor interactions, and energetic contributions, and their trajectories were extended for an additional 500 ns to obtain the final stable binding modes.

### 2.2 Cannabinoid structures

Three-dimensional structures of THC, CBD, THCA, and CBDA were retrieved from the PubChem database (THC, CID 16078; CBD, CID 644019; THCA, CID 98523; CBDA, CID 160570).

### 2.3 PPARγ structures

All PPARγ LBD structures were retrieved from the Protein Data Bank. Potential binding cavities within the PPARγ-apo LBP (PDB ID 1PRG) were identified using Fpocket (Le Guilloux et al., 2009), which detects pockets based on α-sphere clustering. The analysis was performed using the default parameters. Initially, docking calculations were performed against the apo receptor structure (PDB ID: 1PRG) and against the PPARγ/ajulemic acid (AJA, Figure 1) complex (PDB ID: 2OM9). Subsequently, to define the set of 70 structures employed for docking (Supplementary Table S1), a structural similarity analysis of the co-crystallized ligands was conducted using DataWarrior (OpenMolecules, Switzerland) (Sander et al., 2015). Molecular fingerprints were generated using the FragFp descriptor, and pairwise similarities were calculated using the Tanimoto coefficient. Compounds were grouped by hierarchical clustering and subsequently the most complete available structure was selected as representative of each ligand family. Supplementary Table S1 specifies the chain used for each structure and indicates any unresolved regions of the receptor.

### 2.4 Docking

Docking calculations were performed with Autodock4 (Morris et al., 2009). Co-crystallized ligands were removed from the PPARγ LBD structures, and optimized AM1 geometries of THC, THCA, CBD and CBDA were docked into the selected receptors (Supplementary Table S1). Ligand rotatable bonds were treated as flexible, whereas the protein was considered rigid. A rectangular grid of 100 × 100 × 100 points with a 0.375 Å spacing was centered on the ligand-binding pocket to fully cover the three arms of the LBP and all co-crystallized ligands. For each ligand, 1,000 independent docking runs were carried out using the genetic-algorithm search method. Resulting poses were clustered using an RMSD tolerance of 2 Å. The most favorable poses from clusters with population >30% (THC and THCA) or >15% (CBD and CBDA) were subsequently re-clustered using the K-means algorithm and the RMSD of the ligand heavy atoms as the distance metric to obtain five representative clusters for each ligand.

### 2.5 Molecular dynamics simulations

Initial PPARγ/cannabinoid complexes were constructed from the corresponding PDB coordinates and from the docking solutions of the five selected representative clusters. Pose selection prioritized the energy/frequency relationship and receptor structural integrity. Unresolved loops of PPARγ (Supplementary Table S1) were modeled with Modeller 10.5 (Webb and Sali, 2016). All MD simulations were performed with AMBER22 (Case et al., 2022). Protein parameters were taken from the ff14SB force field. Cannabinoid parameters were assigned using the general AMBER force field (GAFF) and RESP charges derived at the HF/6-31G** level. Systems were solvated in an octahedral TIP3P water box and were relaxed and equilibrated prior to production by a solvent minimization followed by a staged heating and multi-step NVT-NPT equilibration scheme. Solvent minimization was carried out with harmonic positional restraints (100 kcal mol^-1^·Å^-2^) on solute heavy atoms using conjugate-gradient minimization. Systems were then heated from 100 K to 298 K over a 1.0 ns NVT segment (1 fs timestep) while maintaining strong positional restraints on protein and ligand atoms (100.0 kcal mol^-1^·Å^-2^). Subsequent equilibration consisted of a series of 1 ns NPT segments (1 fs timesteps) with progressively relaxed restraints over the solute, followed by a final unrestrained NPT segment. SHAKE was applied to constrain bonds involving hydrogen, permitting the employed timesteps; a nonbonded cutoff of 8.0 Å was used during simulations. Pressure (1 atm) was controlled using AMBER’s Monte-Carlo barostat in conjunction with isotropic periodic boundary conditions and temperature was regulated with the Langevin thermostat at 298 K. Production simulations were performed in the NPT ensemble with a 2 fs timestep, continued from the final equilibrated restart, using the same thermostat and barostat settings.

### 2.6 Analysis

MD trajectories were analyzed with the CPPTRAJ software (Roe and Cheatham, 2013). Cluster analysis and representative snapshots of the PPARγ/ligand complexes were obtained with the cluster command in Cpptraj using the hierarchical agglomerative algorithm with average-linkage and the RMSD of the ligand heavy atoms as the distance metric. VMD 1.9.3 was used for visualization and figure preparation (Humphrey et al., 1996). Persistent polar interactions were defined by receptor-ligand heavy-atom distances shorter than 3.5 Å for more than 40% of the simulation time. For aromatic-aromatic interactions, a cutoff distance of 6 Å between the centers of mass of the participating aromatic rings was used. Energetic contributions corresponded to electrostatic (Ele) and Van der Waals (VdW) terms, and binding free energies (ΔGbind) were estimated using the MM/GBSA approach as implemented in the MMPBSA. py module of AmberTools22. The analysis was performed using 200 frames extracted from the last 200 ns of the MD trajectories. Implicit solvent calculations were performed using the Generalized Born (GB) model with the Onufriev-Bashford-Case model and a salt concentration of 0.150 M.

## 3 Results

### 3.1 Characterization of the PPARγ LBP

**FIGURE 3:**
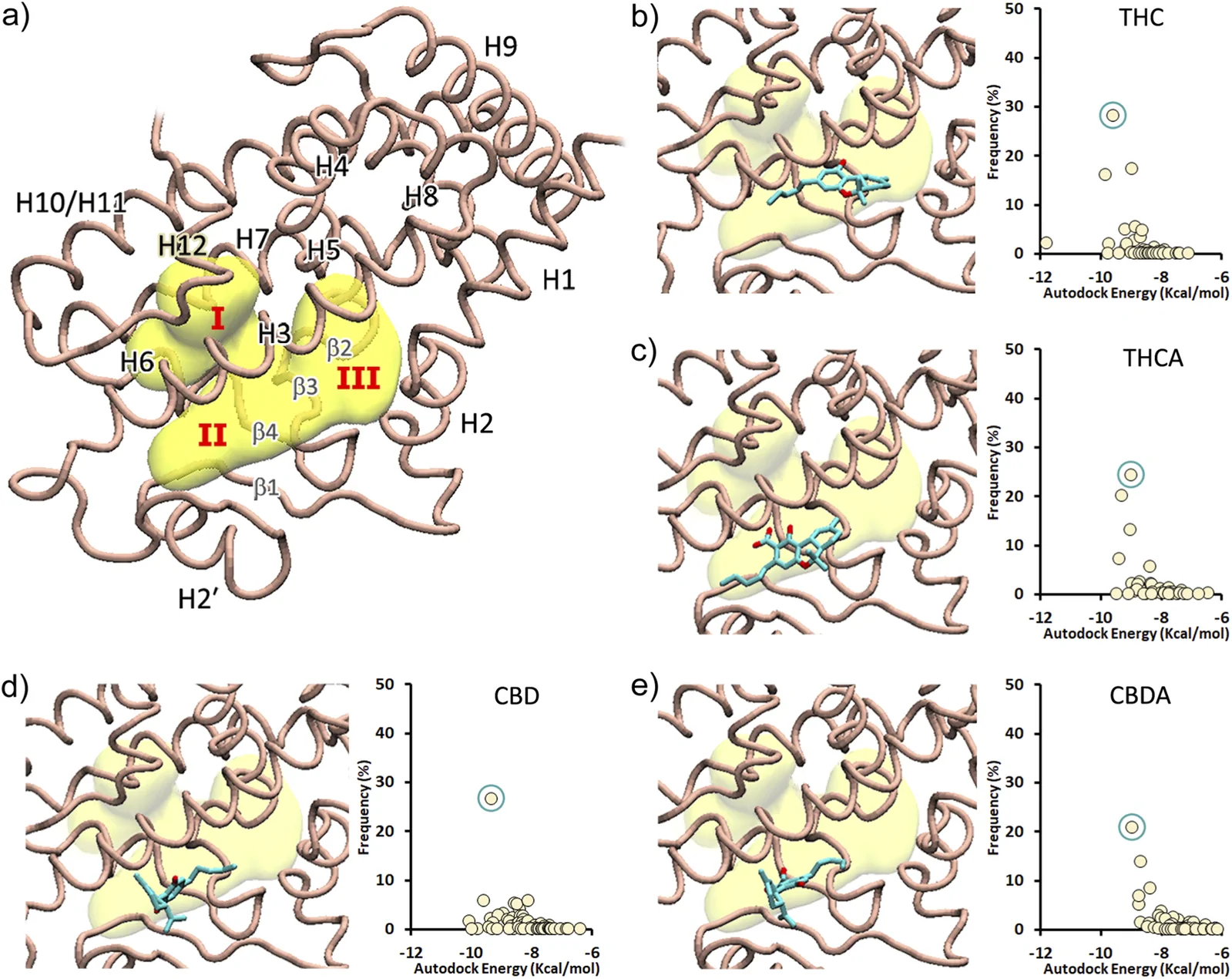
**(a)** Crystal structure of the PPARγ LBD-apo (PDB ID: 1PRG). Secondary structure elements are labeled. The yellow surface represents the cavity detected by Fpocket within the LBP (arms I-III). **(b–e)** Docking of cannabinoids into the PPARγ LBD-apo (PDB ID: 1PRG). Left: lowest-energy solution from the most frequent cluster; right: Autodock4 frequencies and energies for THC **(b)**, THCA **(c)**, CBD **(d)**, and CBDA **(e)**.

The PPARγ LBD consists of 13 α-helices (H1-H12 and the short helix H2’) and a four-stranded β-sheet (β1-β4), arranged in a three-layered antiparallel α-helical sandwich that encloses a large internal LBP with a characteristic Y-shaped geometry. The availability of the PPARγ LBD-apo crystal structure (PDB ID: 1PRG) enabled structural characterization of the LBP in its unliganded state and identification of its three principal arms using the pocket-detection software Fpocket (Figure 3a). Arm I is predominantly lipophilic and comprises residues from H3, H5, H7, H11, the H11-H12 loop, and H12. Arm II is mainly hydrophobic and includes residues from β3/β4, H2’, the H2′-H3 loop, H3, H6, and H7. In contrast, Arm III is formed by residues from the β-sheet, H2, the H1-H2 loop, H3, and H5, and contains both hydrophobic and polar regions. Together, these three interconnected subpockets define the structural framework that accommodates the wide chemical diversity of PPARγ ligands. This structure was then used for the initial Autodock4 docking calculations to explore the possible binding modes of cannabinoids within the PPARγ LBP.

### 3.2 Cannabinoid docking in the PPARγ LBP-apo

A docking grid covering the three arms of the LBP was defined, and 1,000 poses were generated for each ligand using the genetic algorithm. The resulting solutions were clustered using an RMSD cutoff of 2 Å. For each cluster, the lowest binding energy, mean binding energy, and corresponding frequency were determined (Figures 3a–e). Although no highly populated clusters were observed in any case (none exceeded 30% of the total poses), a predominant cluster could be identified for all four ligands. In all cases, this cluster corresponded to binding modes in which the cannabinoid was positioned at the center of the LBP, at the intersection of arms II and III. The mean binding energies of the clusters ranged from −6 to −10 kcal/mol for all cannabinoids, with no significant differences among them. Notably, the presence of the acidic group did not appear to influence ligand orientation in the most populated clusters, as only minimal differences were observed between the poses adopted by THC and THCA, as well as between those of CBD and CBDA. In addition, no clusters with appreciable population were detected in which the cannabinoids occupied alternative regions of the LBP. In light of these results, and to further explore potential cannabinoid binding modes in PPARγ, docking calculations were next performed using the crystal structure of PPARγ in complex with the synthetic THC analog ajulemic acid (AJA) (PDB ID: 2OM9, Supplementary Figure S1).

### 3.3 Cannabinoid docking in the LBP of the PPARγ/ajulemic acid complex

AJA is a synthetic cannabinoid analog that retains the tricyclic scaffold of THC while incorporating a carboxylic group on the A ring and an extended alkyl side chain (Figure 1). To assess whether natural cannabinoids could adopt binding modes similar to those of this synthetic analog, THC and THCA were docked into the PPARγ/AJA complex structure. First, the docking protocol was validated by redocking AJA into the initial crystal structure PDB ID: 2OM9, where the crystallographic pose was reproduced with an RMSD of 1.8 Å, indicating satisfactory agreement between the predicted and experimental binding modes (Supplementary Figure S1a). Then, for the THC and THCA, and consistent with the previous docking results, no highly populated clusters were identified; only in the case of THC did one cluster exceed 35% of the total poses (Supplementary Figure S1b-e). Notably, the most populated THC and THCA clusters displayed binding orientations different from that observed for AJA, although they were highly similar to the poses obtained from docking into the PPARγ LBD-apo. These results suggest that THC and THCA are unlikely to be accommodated within the binding pocket in a binding mode analogous to AJA. Instead, the binding mode observed for AJA was likely driven by the presence of the carboxylic group at a position different from that found in the natural cannabinoids. Based on these results, an alternative strategy was adopted to further explore potential binding modes of natural cannabinoids within PPARγ.

### 3.4 Cannabinoid docking in a set of 70 structures of the PPAR LBP-holo

Considering the large number of ligand-bound PPARγ structures available, with more than 250 entries deposited in the Protein Data Bank, a more comprehensive approach was employed by performing docking calculations of the cannabinoids across a curated set of PPARγ structures co-crystallized with structurally diverse ligands. Given the intrinsic flexibility of the binding pocket and the structural variability of the co-crystallized ligands, this strategy was expected to exploit ligand-induced expansions of the subcavities, thereby enabling the identification of representative cannabinoid binding poses distinct from those observed in the PPARγ LBD-apo structure.

A manual selection of available PPARγ structures was performed, avoiding redundancy of highly similar ligands and ensuring the inclusion of complexes displaying ligand atoms spanning all LBP arms. This resulted in an initial dataset comprising 70 PPARγ structures (Supplementary Table S1). Structural superposition of these complexes illustrates the chemical diversity of ligands considered and their distribution across the different arms of the LBP (Supplementary Figure S2). Docking of the four natural cannabinoids was then carried out under the same conditions previously applied to the PPARγ-apo and PPARγ/AJA structures.

**FIGURE 4:**
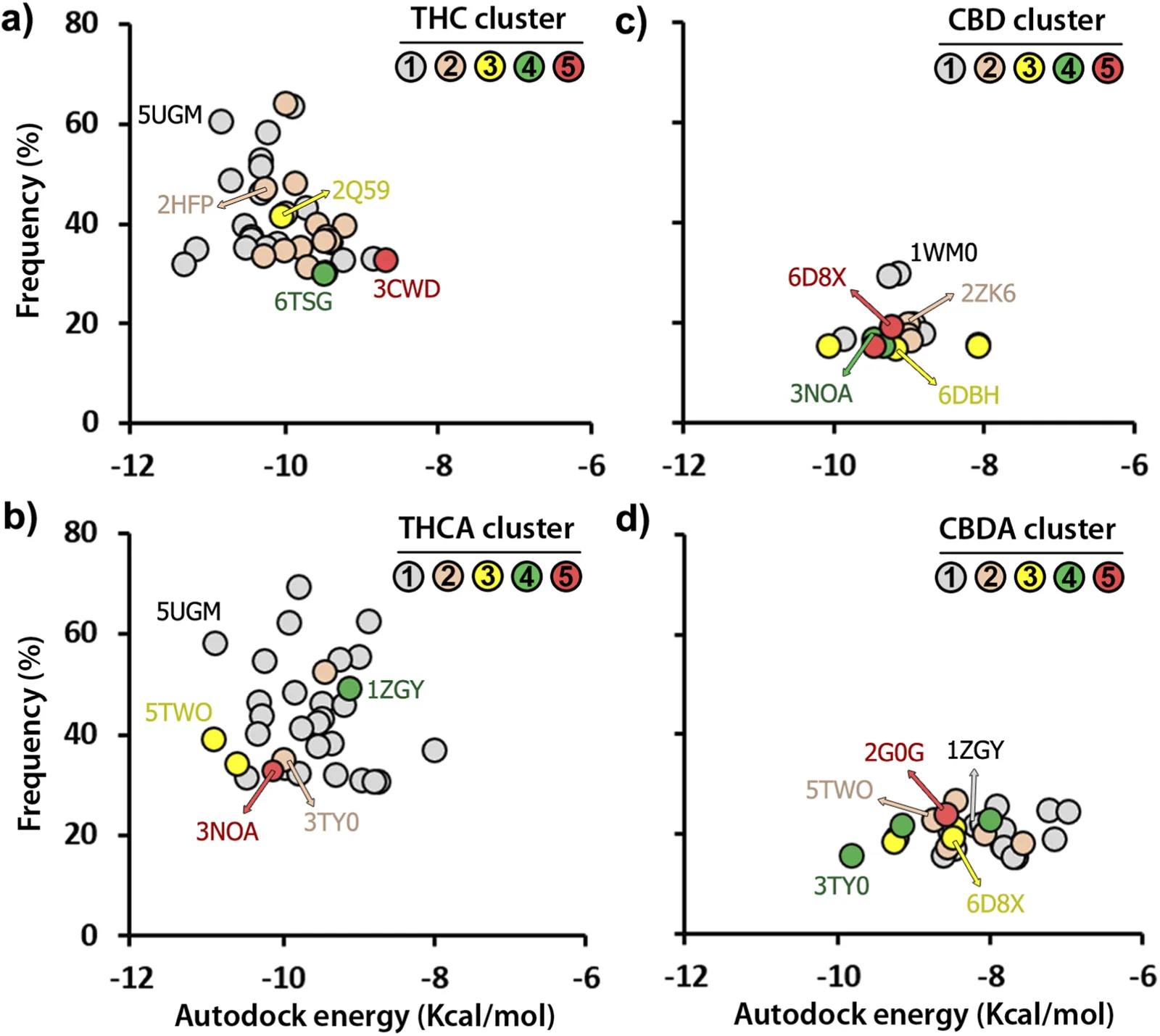
Autodock4 frequencies and energies for the docking solutions of THC **(a)**, THCA **(b)**, CBD **(c)**, and CBDA **(d)** across 70 PPARγ LBD-holo structures, reclustered by the ligand RMSD. The PDB IDs of the five structures selected for MD simulations are indicated.

**FIGURE 5:**
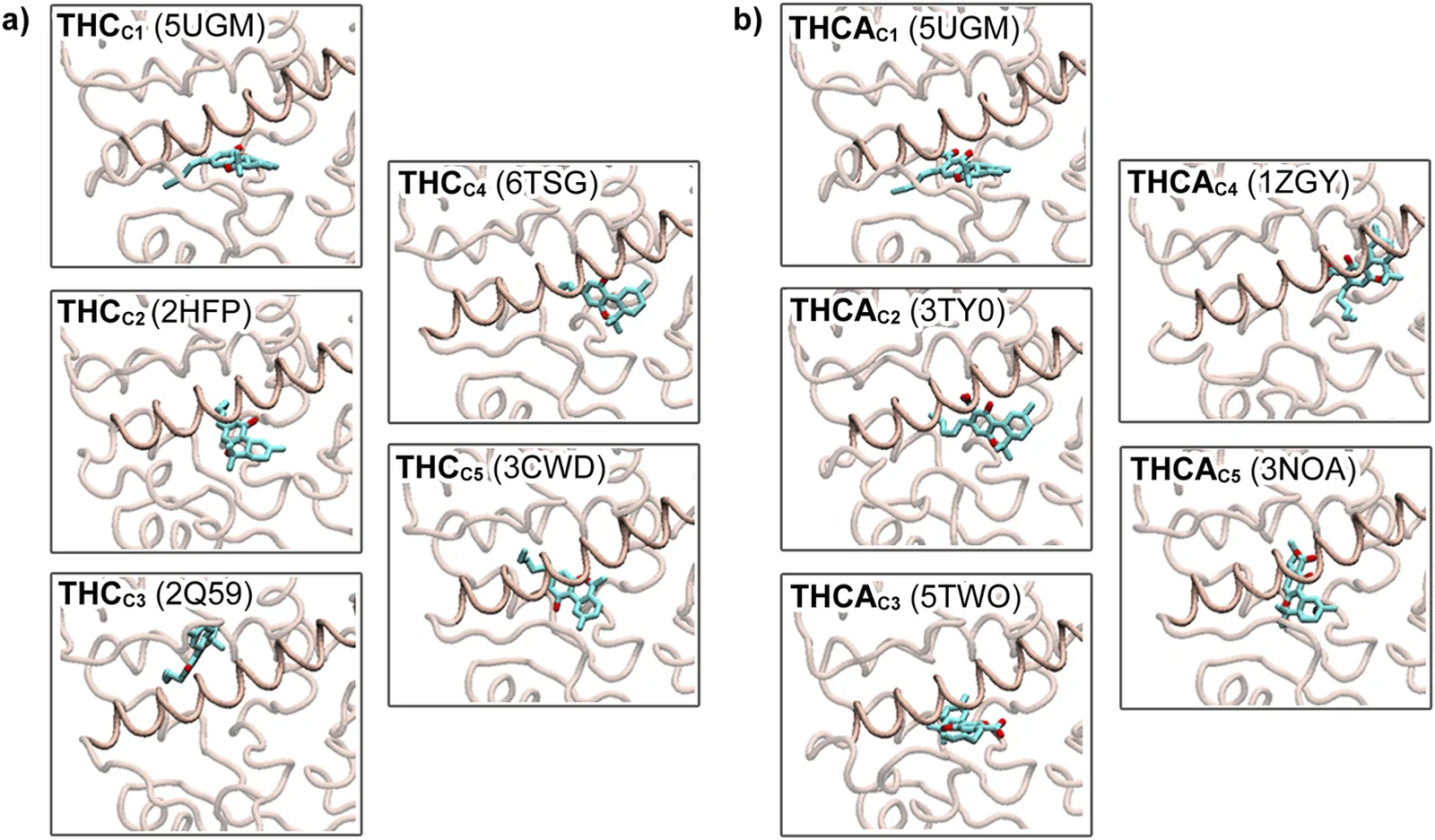
Selected docking solutions for THC **(a)** and THCA **(b)**, each representative of one of the five clusters identified after RMSD-based reclustering of all solutions with a frequency greater than 30%.

**FIGURE 6:**
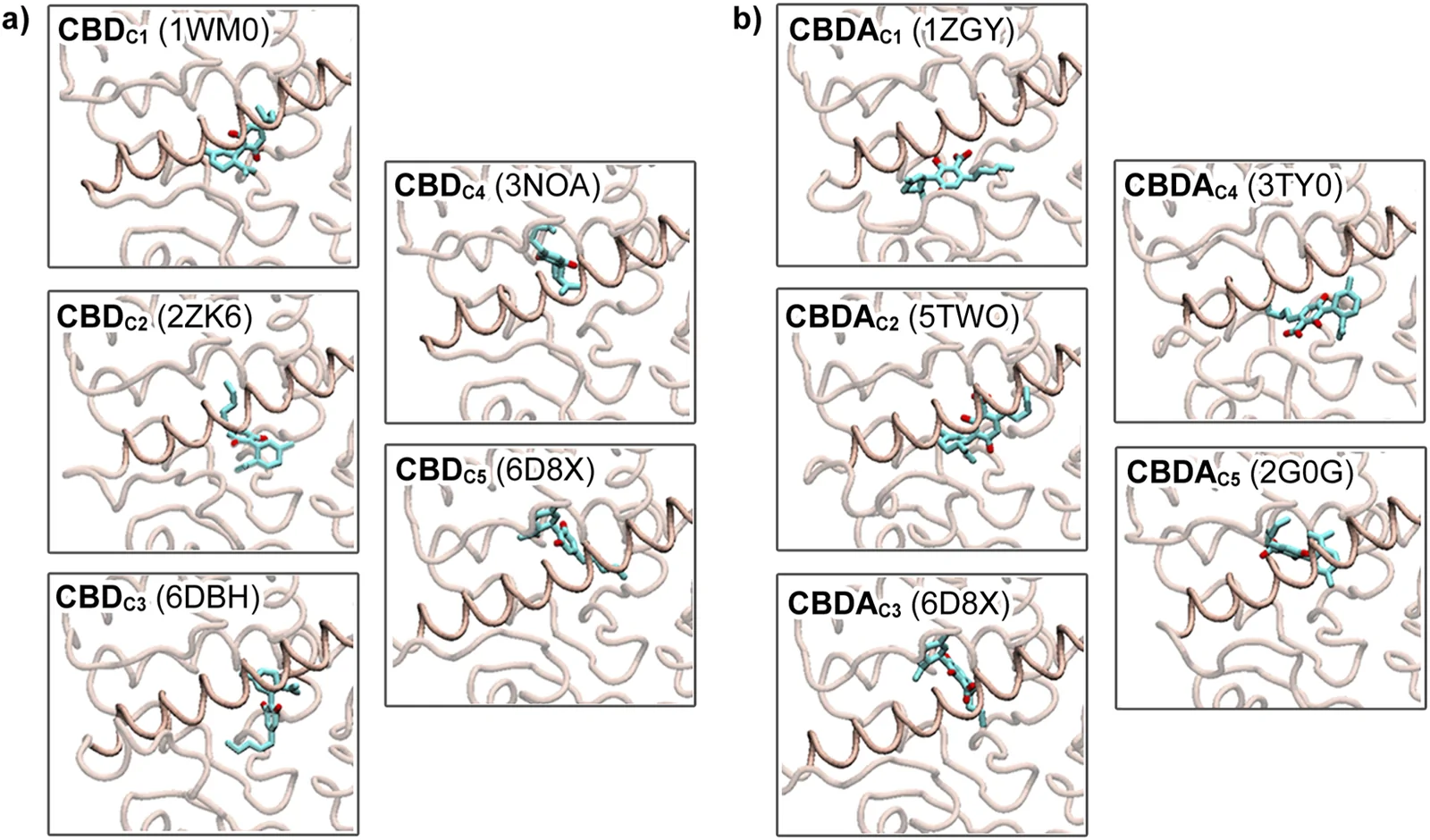
Selected docking solutions for CBD **(a)** and CBDA **(b)**, each representative of one of the five clusters identified after RMSD-based reclustering of all solutions with a frequency greater than 15%.

As a first criterion for analysis, a frequency threshold of 30% was established for THC and THCA clusters, and 15% for CBD and CBDA, since the cluster populations obtained for the latter two cannabinoids were lower, likely due to their greater conformational flexibility relative to the more rigid fused-ring system of THC and THCA. Under these criteria, 37 solutions for THC, 32 for THCA, 18 for CBD, and 27 for CBDA satisfied the defined threshold (Supplementary Table S2). The lowest-energy pose from each cluster for each ligand was then reclassified into five groups based on heavy-atom RMSD in order to group structurally similar solutions. Figure 4 shows the frequency-energy plots colored according to the clusters assigned after RMSD-based reclustering. A representative binding mode from each of the five clusters for each ligand was selected and is shown in Figures 5, 6.

#### 3.4.1 THC

The 37 THC solutions were primarily grouped into two major clusters: THC_C1_, comprising 20 solutions similar to the most frequent pose obtained for apo PPARγ, and THC_C2_, comprising 14 solutions in which the cannabinoid adopted an alternative arrangement, positioned slightly deeper within arm III. The remaining three clusters (THC_C3_, THC_C4_ and THC_C5_) each contained a single solution. In THC_C3_, the ligand was located entirely within arm I; in THC_C4_, within arm III, but in a configuration that differed from THC_C2_; and in THC_C5_, again at the intersection between arms II and III, although with a different orientation from that observed in THC_C1_.

#### 3.4.2 THCA

Reclustering of the THCA solutions likewise revealed a predominant cluster (THCA_C1_, 26 out of 32 solutions), whose pose, as observed for THC, resembled that obtained from docking to PPARγ-apo. The remaining clusters were considerably less populated (with two solutions each for THCA_C2_ and THCA_C3_, and a single solution for THCA_C4_ and THCA_C5_). In THCA_C2_, THCA_C3_, and THCA_C5_, the cannabinoid occupied the center of the LBP, although with different orientations. In contrast, in THCA_C4_ the ligand was inserted exclusively within arm III of the LBP.

#### 3.4.3 CBD

Fewer CBD solutions reached the frequency threshold, and reclustering yielded three clusters (CBD_C1_ with 6 solutions, CBD_C2_ with 5 solutions, and CBD_C3_ with 3 solutions) in which the cannabinoid occupied the region defined by the intersection of the three arms of the LBP. None of these solutions resembled the pose obtained for PPARγ-apo. The remaining two clusters (CBD_C4_ and CBD_C5_) each contained two solutions and corresponded to poses in which CBD was located within arm I.

#### 3.4.4 CBDA

In the most populated CBDA cluster (CBDA_C1_, 14 out of 27 solutions), the cannabinoid adopted the same overall position as THCA in THCA_C1_, with the carboxylate group oriented toward H6, although the side chain and A ring were arranged in opposite orientations. In CBDA_C2_ (6 solutions) and CBDA_C4_ (3 solutions), the cannabinoid was inserted predominantly into arm III, whereas in CBDA_C3_ (3 solutions) and CBDA_C5_ (1 solution) it occupied arm I.

In summary, our docking strategy applied to 70 holo PPARγ structures allowed us to identify different binding modes, many of them displaying high population values that were not obtained in the docking performed on PPARγ-apo. In addition to the poses located at the intersection between arms II and III, where the cannabinoids could adopt alternative orientations, solutions in which the ligand predominantly occupied either arm I or arm III were also detected. Thus, by selecting five representative solutions from the clusters obtained for each ligand, an initial set of 20 PPARγ/cannabinoid complexes was constructed and subsequently used as starting points to evaluate the stability of the different binding modes through MD simulations.

### 3.5 Molecular dynamics simulation of PPARγ/cannabinoid complexes

#### 3.5.1 Ligand stability and principal component analysis

In order to evaluate the stability of the poses previously identified by docking, receptor-ligand complexes were constructed and immersed in an octahedral box of TIP3P water molecules, and three independent 500 ns MD simulation replicates were carried out for each system. As an initial approach to assess the dynamic behavior of the cannabinoids in the simulated systems, the position of the ligand center of mass was monitored throughout each trajectory (Supplementary Figures S3 and S4). This analysis showed that the cannabinoids occupied different regions of the LBP, with ligand-dependent behaviors, and that in most systems, the ligand position was conserved across replicates, although a pronounced displacement from the initial location was observed in some cases (e.g., 2HFP/THC, 3NOA/THCA, and 3TY0/CBDA).

**FIGURE 7:**
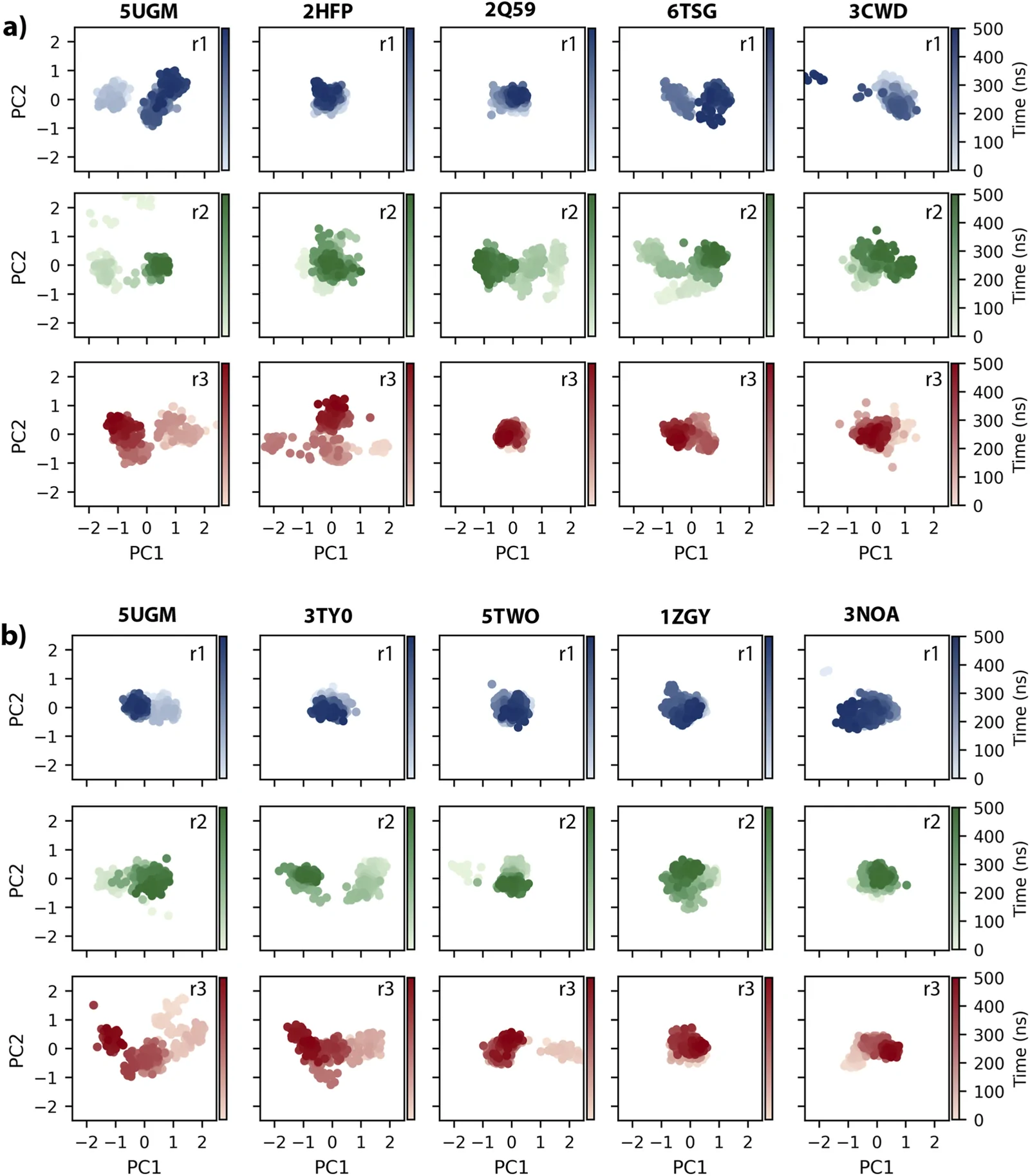
Principal component analysis (PCA) for THC **(a)** and THCA **(b)** trajectories. Plots of the projection of ligand coordinates onto the first and second eigenvectors (PC1 vs. PC2). Points are colored according to simulation time from lightest (0 ns) to darkest (500 ns).

**FIGURE 8:**
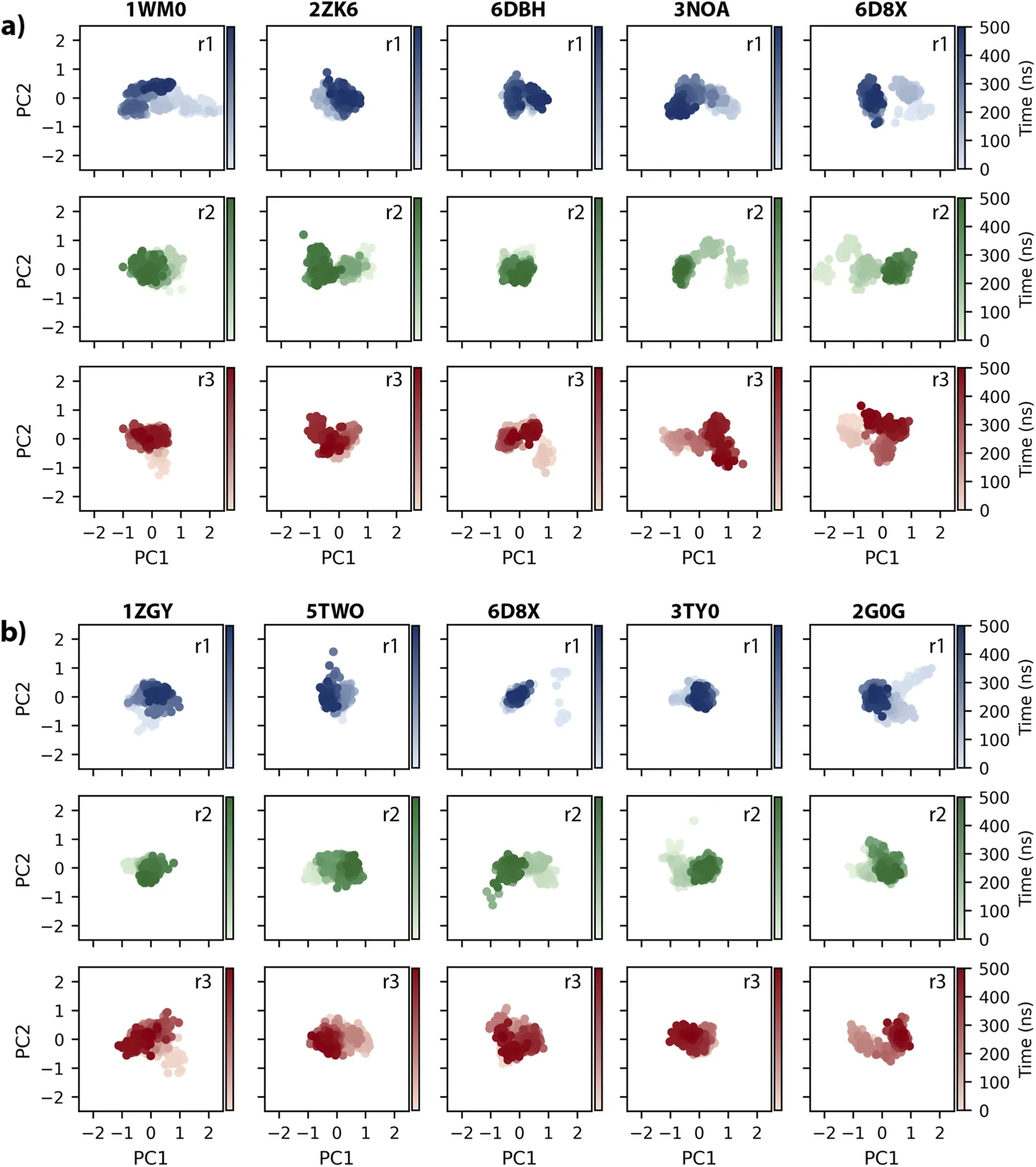
Principal component analysis (PCA) for CBD **(a)** and CBDA **(b)** trajectories. Plots of the projection of ligand coordinates onto the first and second eigenvectors (PC1 vs. PC2). Points are colored according to simulation time from lightest (0 ns) to darkest (500 ns).

To simplify the analysis, the dimensionality of the problem was reduced by performing a principal component analysis (PCA), obtaining the eigenvectors and plotting the projection of the main modes (PC1 vs. PC2) over simulation time (Figures 7, 8). This approach revealed different types of behavior, ranging from systems in which the cannabinoid remained essentially in the same position throughout the simulation to others in which the ligand did not adopt a stable binding mode along the trajectory. In some cases, the ligand showed substantial movement during the first nanoseconds and then stabilized in a defined position. Conversely, there were systems in which the ligand appeared stable at the beginning but later lost that stability as the simulation progressed. To quantify the overall variation in ligand mobility, the variance of the point distribution in the PC1 vs. PC2 plots was calculated, which allowed us to identify the most stable systems for each ligand (Supplementary Figure S5).

Pairwise ligand RMSD analysis also helps assess whether the cannabinoid binding mode converges, as it reflects the similarity of its configuration over time (Supplementary Figures S6 and S7). These plots allow us to distinguish between systems that reach stability (characterized by low RMSD values away from the diagonal) and those showing higher and more variable RMSD values, indicative of transitions between or sampling of multiple conformations. Notably, the acidic cannabinoids exhibit a greater number of stable binding modes than THC and CBD, likely because the presence of the carboxylic group enables stronger interactions with residues lining the PPARγ LBP.

#### 3.5.2 Analysis of polar interactions between PPARγ and cannabinoids

In the next stage of the analysis, the formation of polar interactions (hydrogen bonds and salt bridges) between the cannabinoids and PPARγ residues was examined across the 60 simulated trajectories. The number of persistent interactions, defined as those with an occupancy greater than 40% along the simulation, identified in each system is provided in Supplementary Figure S5. By monitoring the time evolution of the distances between the atoms involved in these interactions (Supplementary Figures S8-S11), it was observed that although in a few cases interactions present at the beginning were subsequently lost, in most systems they remained stable throughout the trajectory or, once formed, were maintained until the end of the simulation. For all four cannabinoids, there were systems in which frequent hydrogen bonds were established, stabilizing the ligand in a defined conformation. In addition, the acidic cannabinoids formed highly stable salt bridges in several systems with basic residues within the LBP, namely, Arg280 (H3), Arg288 (H3), and Lys367 (H7) (Supplementary Figure S12). As a result, the overall number of polar interactions was higher for the acidic cannabinoids than for THC and CBD.

To select the most stable binding modes, only those systems displaying at least one stable polar interaction during the last 300 ns of the simulation were selected for further analysis. This subset was expanded by including five additional systems (2HFP/THC/r1, 3TY0/THCA/r1, 1ZGY/THCA/r3, 2ZK6/CBD/r1, and 6DBH/CBD/r2), which were of particular interest because, although they did not exhibit persistent hydrogen bonds, they showed very low mobility throughout the entire simulation (Supplementary Figure S5). Supplementary Table S3 summarizes the systems selected for further analysis: 8 systems for THC grouped into 6 binding modes according to the average position and orientation of the cannabinoid, 11 systems for THCA grouped into 7 binding modes, 8 systems for CBD classified into 5 modes, and 13 systems for CBDA distributed among 5 binding modes.

#### 3.5.3 Analysis of aromatic-aromatic interactions between PPARγ and cannabinoids

To evaluate the formation of aromatic interactions, the distance between the center of the cannabinoid resorcinol ring and the centers of the aromatic side chain rings of the amino acid residues, together with the angle between their respective normal vectors, was monitored throughout the simulations. These parameters were represented in a 2D histogram, allowing us to assess the stability of the identified interactions (Supplementary Figure S13). Four LBP residues were found to form this type of interaction persistently in several systems: Phe282 (H3), Phe287 (H3), Tyr327 (H5), and Phe363 (H7) (Supplementary Figure S12; Supplementary Table S3). In the case of THC, two systems were particularly stable: in 2HFP r1, a stable interaction was established with Tyr327, whereas in 2Q59 r3 similar contacts were formed with two residues, Phe282 and Phe363. In 2Q59 r1, the same interactions were observed as in 2Q59 r3, although they were less stable. By contrast, CBD formed aromatic interactions of moderate stability with Tyr327 in two systems, 3NOA r1 and 6D8X r1, while for CBDA, the interaction with Tyr327 was highly stable in three systems, 6D8X r1 and r2, and 2G0G r2. No persistent aromatic interactions were observed for THCA.

#### 3.5.4 Representative binding modes

To obtain a representative MD structure for each of the binding modes listed in Supplementary Table S3, the frames corresponding to the last 300 ns of each trajectory were clustered. In all cases, a predominant cluster was identified, with frequencies consistently above 80% (Supplementary Table S3). Supplementary Figures S14-S17 compile these binding modes, highlighting the residues involved in cannabinoid recognition through polar or aromatic-aromatic interactions.

##### 3.5.4.1 THC

Six binding modes were identified for THC (Supplementary Figure S14). In modes 1a and 1b, the overall orientation of THC is similar; however, in the first mode the ether oxygen interacts with Ser342, whereas in the second the hydroxyl group interacts with Ile281 (H3). In mode 2, the hydroxyl group forms a contact with Cys285 (H3), and Tyr327 also contributes to stabilization. In binding mode 3, the ligand hydroxyl interacts with Leu340. Two systems converged toward binding mode 4, in which the THC hydroxyl interacts with two residues from H3 (Phe282 and Gln286), while Phe282 and Phe363 establish aromatic contacts with the resorcinol ring. In binding mode 5, the ether oxygen of THC is oriented toward His449 in H12, and the resorcinol ring again contacts Phe363. Finally, in mode 6, the backbone carbonyl of Arg288 interacts with the hydroxyl group of THC.

##### 3.5.4.2 THCA

Seven binding modes were identified for THCA (Supplementary Figure S15). In modes 1a and 1b, the carboxylate group forms a salt bridge with Arg280, while the ether oxygen establishes a hydrogen bond with the backbone of Ser342. The difference between these two modes lies in the additional contact formed in mode 1a, where the hydroxyl group also interacts with the backbone of Ile281. Modes 2 and 6 do not display either polar or aromatic interactions between ligand and receptor. Two replicates converge toward mode 3, in which the carboxylate interacts with the backbone of Ser342. Mode 4, which resembles mode 3, involves an additional contact between the carboxylate and Arg288, while the hydroxyl group interacts with Glu343. Mode 7 also shows an interaction between the carboxylate and the Arg288/Ser342 pair, although the cannabinoid adopts a different orientation. Finally, three systems converge toward mode 5, all characterized by interaction between the THCA carboxylate and the Lys367/Tyr327 pair. In mode 5b, Cys285 and Ser289 also contribute to ligand recognition, whereas in mode 5a the cannabinoid interacts with Gln286.

##### 3.5.4.3 CBD

Five binding modes were identified for CBD (Supplementary Figure S16). In binding mode 1, one of the cannabinoid hydroxyl groups interacts with Ser289 (H3). Two systems converge toward binding mode 2a, in one of which a hydrogen bond is formed with Leu340. In binding mode 2b, the overall position of the cannabinoid is similar, but interactions are established with two H3 residues, Ile281 and Cys285. In binding mode 3, although CBD is also oriented toward H3, no polar interactions with the protein are observed. In contrast, in binding mode 4, two residues, Phe363 and Tyr327, contact the same hydroxyl group. In the two systems converging toward binding mode 5, a contact with Ser289 (H3) is observed; however, they differ in the interaction formed by the second hydroxyl group, which involves Tyr327 in one case and His449 in the other.

##### 3.5.4.4 CBDA

Although several systems with stable ligand positioning were identified for CBDA, they can be grouped into only five binding modes (Supplementary Figure S17). In binding mode 1a, the cannabinoid carboxylate interacts with Ser342; additionally, Asp260 and Phe287 contribute to recognition through contacts with the ligand hydroxyl groups. In binding mode 1b, CBDA adopts a highly similar orientation, but instead interacts with Arg288 and Ile267. Five systems converge toward a binding mode in which the cannabinoid adopts an analogous configuration. In mode 2a, an interaction with Ser342 is established, in this case through one of the ligand hydroxyl groups. The remaining modes (2b-2d) are characterized by the formation of a salt bridge between Lys367 and the cannabinoid carboxylate, in some cases further complemented by an interaction with Tyr327 (modes 2c and 2d). In mode 2d, an additional contact with Ser289 is formed through one of the hydroxyl groups. In binding mode 3, a hydrogen bond is observed between one hydroxyl group and Tyr327, and between Ser289 and the other hydroxyl group. In one of these systems, Arg288 also participates by forming a salt bridge with the CBDA carboxylate. The backbone atoms of Ser342 and Glu343 further contribute to ligand recognition by contacting the carboxylate group in the binding mode 4. Finally, binding mode 5 comprises a single system and is characterized by the formation of multiple interactions: Arg288 and Ser342 contact the carboxylate group, while Ile281 and Cys285 interact with the para-hydroxyl group.

#### 3.5.5 Analysis of the energetic contributions

To estimate the energetic contributions of the binding modes corresponding to the systems listed in Supplementary Table S3, the MM/GBSA approach was applied to calculate the van der Waals (VDW) and electrostatic (ELE) terms in vacuum, together with the total ΔGbind, which includes the solvation contributions of both ligand and receptor (Supplementary Figure S18). Although some differences between systems were observed, the VDW contribution did not vary substantially, with all values falling within the range of −40 to −55 kcal/mol. On average, CBD displayed the least favorable VDW contribution values. In contrast, for the ELE contribution, a marked difference was observed between systems involving acidic cannabinoids that form salt bridges and those in which the ligand establishes only hydrogen bonds.

By jointly considering the MM/GBSA results and the number and persistence of both polar and aromatic-aromatic interactions between the ligand and the receptor, three systems per ligand were selected for trajectory extension to 1,000 ns, in order to evaluate binding mode stability on this longer timescale.

#### 3.5.6 Extension of selected PPARγ/cannabinoid MD trajectories

**FIGURE 9:**
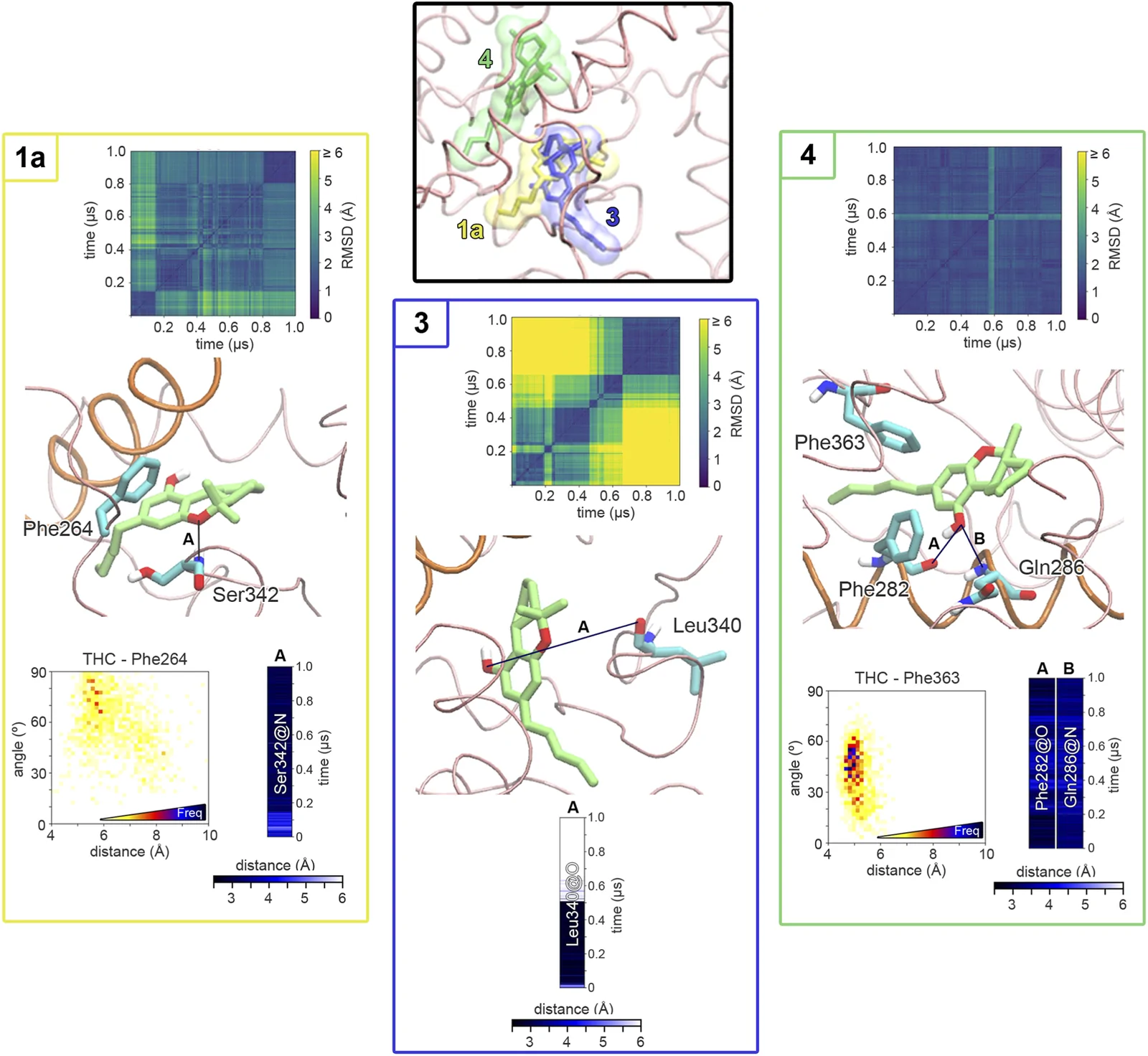
Binding modes of THC from the 1,000 ns trajectories. The top central panel shows the global location of each of the three evaluated binding modes. For each mode, the corresponding panels displayed: (top) pairwise ligand RMSD; (center) a representative snapshot highlighting the residues involved in cannabinoid recognition; and (bottom) a 2D histogram analysis of aromatic-aromatic interactions (where present), showing the joint distribution of the angle between the normal vectors of the aromatic rings (cannabinoid resorcinol ring and side chains of the indicated aromatic residues) and the distance between their centers of mass, alongside the time evolution of the distances between the atoms involved in polar interactions.

**FIGURE 10:**
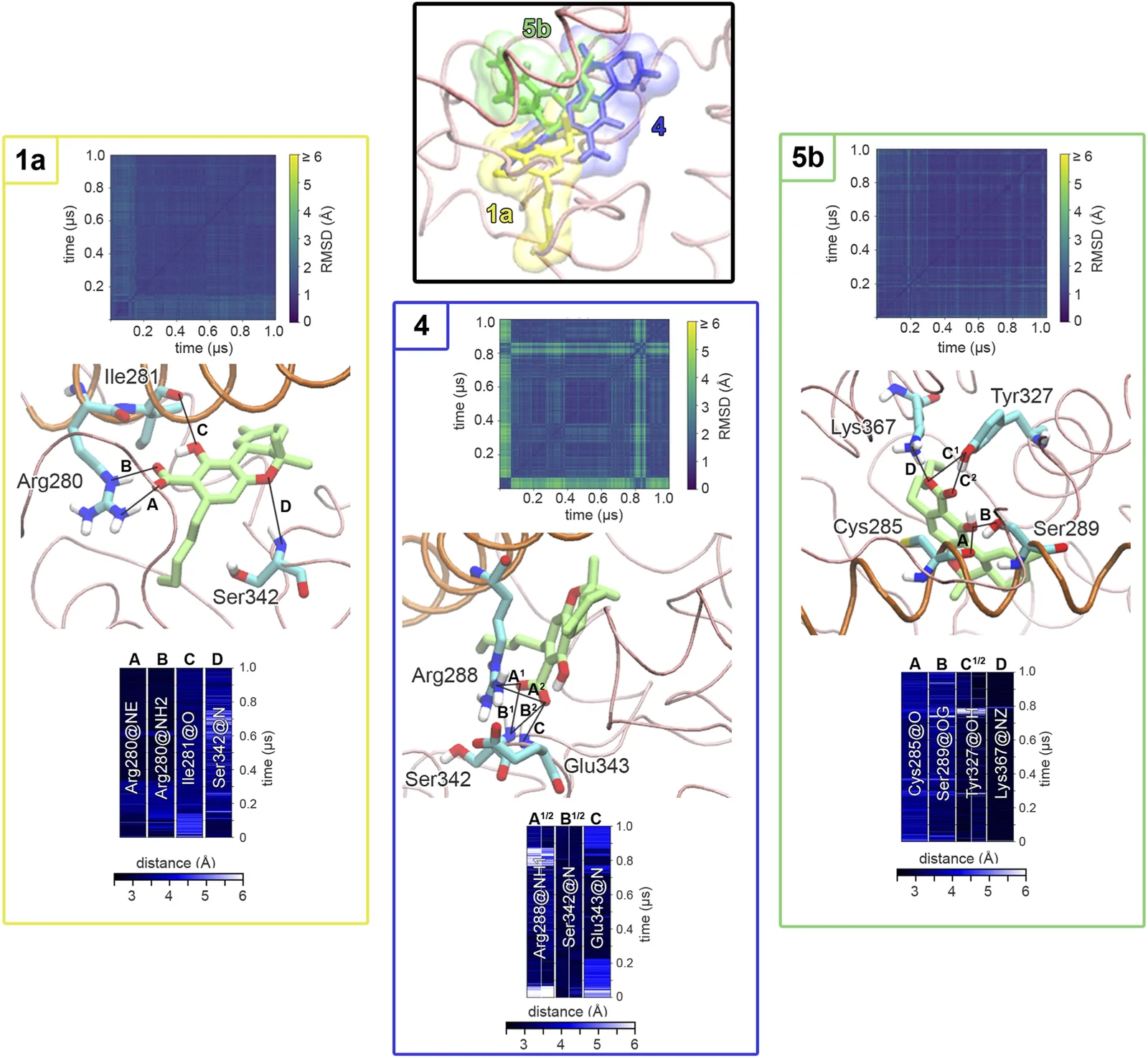
Binding modes of THCA from the 1,000 ns trajectories. The top central panel shows the global location of each of the three evaluated binding modes. For each mode, the corresponding panels displayed: (top) pairwise ligand RMSD; (center) a representative snapshot highlighting the residues involved in cannabinoid recognition; and (bottom) the time evolution of the distances between the atoms involved in polar interactions.

**FIGURE 11:**
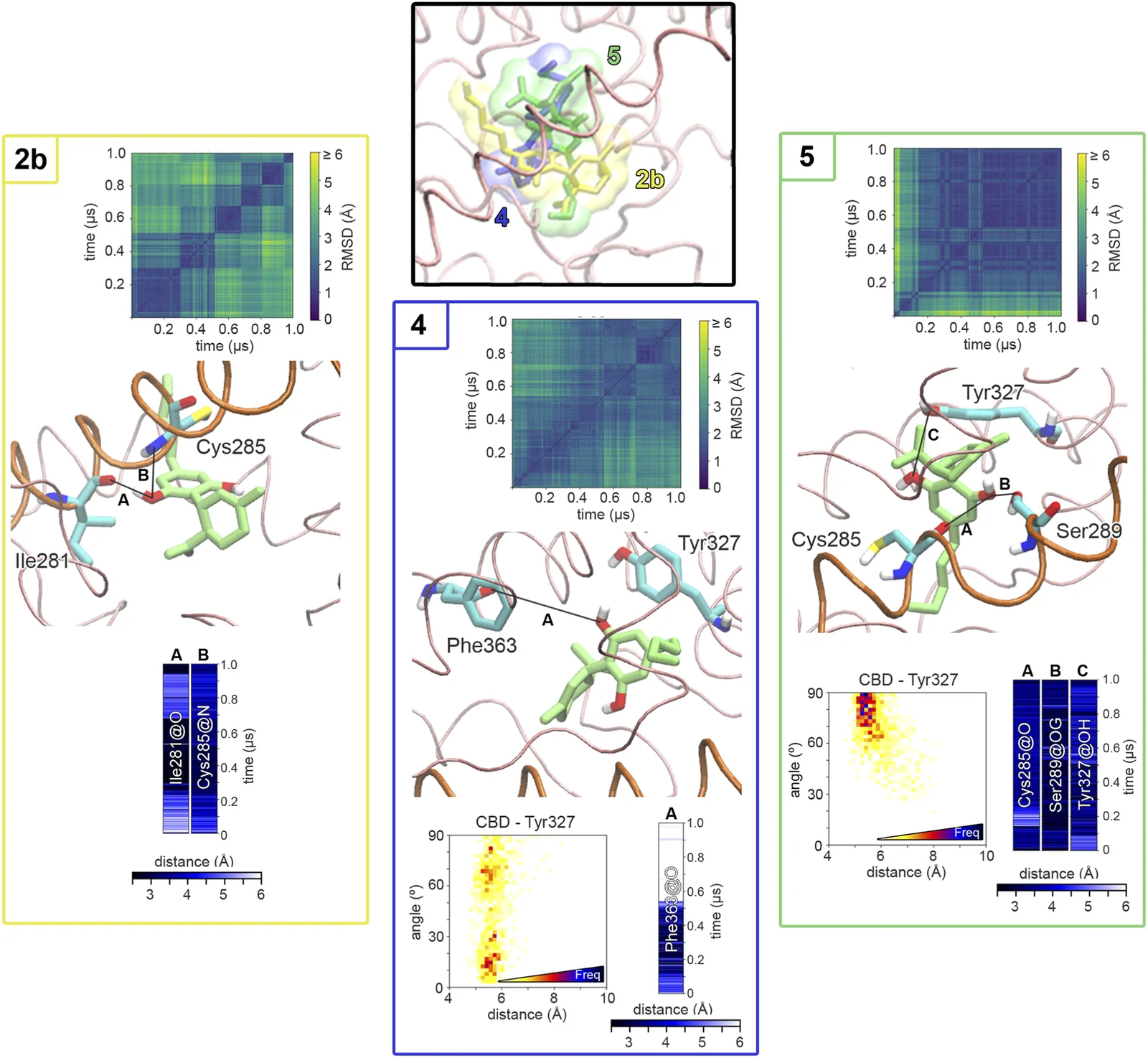
Binding modes of CBD from the 1,000 ns trajectories. The top central panel shows the global location of each of the three evaluated binding modes. For each mode, the corresponding panels displayed: (top) pairwise ligand RMSD; (center) a representative snapshot highlighting the residues involved in cannabinoid recognition; and (bottom) a 2D histogram analysis of aromatic-aromatic interactions, where present, showing the joint distribution of the angle between the normal vectors of the aromatic rings (cannabinoid resorcinol ring and side chains of the indicated aromatic residues) and the distance between their centers of mass, alongside the time evolution of the distances between the atoms involved in polar interactions.

**FIGURE 12:**
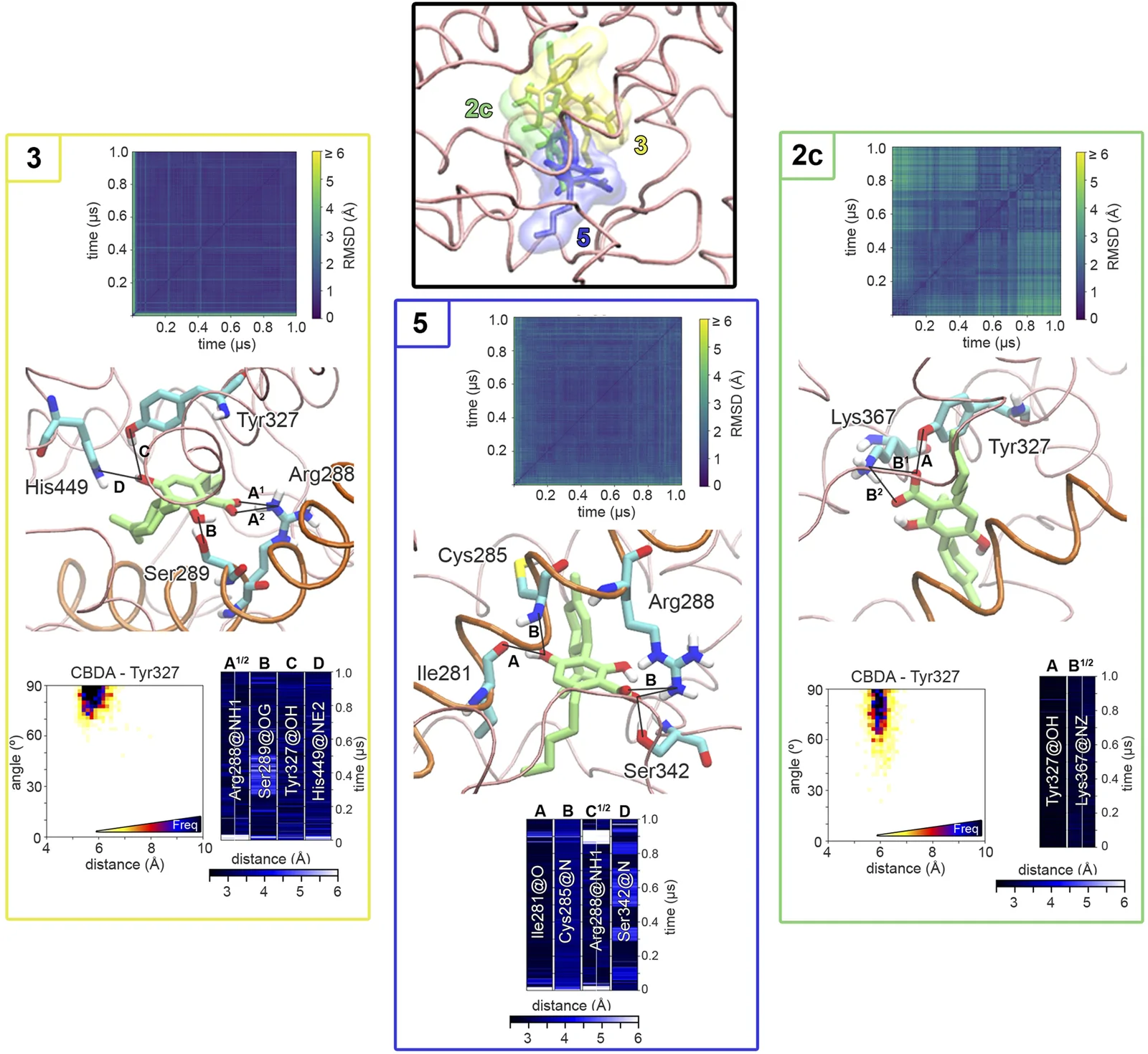
Binding modes of CBDA from the 1,000 ns trajectories. The top central panel shows the global location of each of the three evaluated binding modes. For each mode, the corresponding panels displayed: (top) pairwise ligand RMSD; (center) a representative snapshot highlighting the residues involved in cannabinoid recognition; and (bottom) a 2D histogram analysis of aromatic-aromatic interactions, where present, showing the joint distribution of the angle between the normal vectors of the aromatic rings (cannabinoid resorcinol ring and side chains of the indicated aromatic residues) and the distance between their centers of mass, alongside the time evolution of the distances between the atoms involved in polar interactions.

Table 1 lists the systems selected for each ligand whose trajectories were extended to 1,000 ns? Analysis of the extended trajectories included the determination of pairwise RMSD throughout the entire simulation, monitoring of the time evolution of polar interactions, and monitoring of the formation of aromatic-aromatic interactions between the receptor and the ligand (Figures 9–12). MM/GBSA was also applied to evaluate the energetic contributions in each case (Table 1). Additionally, the contribution of residues involved in polar interactions with the ligands was evaluated through per-residue MM/GBSA energy decomposition and hydrogen-bond occupancy (HBO) analyses (Supplementary Table S4).

**TABLE 1:** Summary of extended (1,000 ns) simulation systems, ligand RMSD, and MM/GBSA values (average ±SD).

					MM/GBSA		
Ligand	Receptor	Run	Mode	RSMD (Å)	ΔE_VDW_	ΔEE_Ele_	ΔG_bind_
THC	5UGM	r1	1a	2.8 ± 0.8	−48.5 ± 0.1	−7.5 ± 0.1	−38.6 ± 0.2
THC	2HFP	r3	3	5.2 ± 1.9	nd	nd	nd
THC	2Q59	r3	4	1.5 ± 0.3	−49.8 ± 0.1	−9.2 ± 0.1	−41.9 ± 0.2
THCA	5UGM	r1	1a	2.1 ± 0.1	−50.4 ± 0.1	−49.2 ± 0.6	−50.3 ± 0.2
THCA	5TWO	r3	4	3.2 ± 0.7	−49.4 ± 0.1	−28.2 ± 0.8	−47.6 ± 0.3
THCA	3NOA	r2	5b	1.7 ± 0.2	−51.0 ± 0.1	−46.1 ± 0.4	−46.4 ± 0.2
CBD	2ZK6	r2	2b	2.6 ± 1.0	−45.9 ± 0.1	−6.2 ± 0.2	−40.6 ± 0.2
CBD	3NOA	r1	4	3.2 ± 0.4	nd	nd	nd
CBD	6D8X	r1	5	3.7 ± 0.7	−47.3 ± 0.1	−7.8 ± 0.2	−40.7 ± 0.2
CBDA	6D8X	r1	3	3.6 ± 0.3	−49.8 ± 0.1	−28.0 ± 0.4	−47.4 ± 0.2
CBDA	3TY0	r2	5	4.0 ± 0.5	−47.9 ± 0.2	−46.2 ± 0.8	−52.8 ± 0.3
CBDA	2G0G	r2	2c	2.6 ± 0.7	−44.3 ± 0.1	−51.2 ± 0.4	−51.4 ± 0.2

All binding modes displayed good stability for the acidic cannabinoids THCA and CBDA, although among these, certain systems were distinguished by their more tightly anchored ligands and more persistent interactions. For THC and CBD, certain modes proved less stable, and in some cases, the interactions anchoring the cannabinoid were lost over the course of the simulation.

##### 3.5.6.1 THC

The most stable binding mode for THC (Figure 9) was binding mode 4, in which the cannabinoid occupies predominantly arm I of the LBP. THC is anchored through its hydroxyl group via persistent polar interactions with the H3 backbone (residues Phe282 and Gln286). A stable aromatic-aromatic interaction between the resorcinol ring and the side chain of Phe363 also contributes to stabilization. In this binding mode, the ligand remains essentially stable throughout the 1,000 ns simulation (as indicated by the absence of high RMSD values off the diagonal) and displays the most favorable ELE contribution and ΔGbind values as estimated by MM/GBSA (Table 1).

Binding mode 1a is slightly less stable, with THC exhibiting greater mobility at the arms II/III intersection, despite the interaction with Ser342 being maintained throughout the simulation. This behavior may be associated with the lower frequency of formation of the aromatic-aromatic interaction with Phe264, which would prevent the cannabinoid from being simultaneously anchored at two distinct points. Both the ELE contribution and ΔGbind are slightly less favorable than in binding mode 4 (Table 1).

Binding mode 3, also located at the arms II/III intersection, proved highly unstable, with THC acquiring substantial mobility and sampling different regions of the LBP. The interaction initially observed between the hydroxyl group and Leu340 was lost beyond 500 ns?

##### 3.5.6.2 THCA

All three binding modes of this cannabinoid remained stable throughout the 1,000 ns simulation (Figure 10), although the pairwise RMSD plots for modes 1a and 5b are appreciably more uniform and exhibit lower RMSD values than those of mode 4, indicating greater conservation of the cannabinoid configuration. In all cases, multiple salt bridges are observed between the carboxylate group and a basic residue of the LBP, which are complemented by additional polar interactions that anchor the cannabinoid at several points simultaneously. No aromatic-aromatic interactions were identified in any of the three modes.

In mode 1a, with THCA located predominantly in arm II, the interaction between the carboxylate group and the guanidinium moiety of Arg280 is dominant and is complemented by a contact between the adjacent hydroxyl and the backbone of Ile281. On the opposite side, the cannabinoid is anchored by an interaction between Ser342 and the ether oxygen of THCA.

In mode 4 (arm III), Ser342 also participates; its backbone atoms, together with those of Glu343, directly contact the THCA carboxylate group. The carboxylate further interacts with the guanidinium group of Arg288, albeit in a geometry less favorable for salt bridge formation than in mode 1a, as reflected by the lower ELE contribution estimated by MM/GBSA relative to mode 1a.

In binding mode 5b, with the cannabinoid located predominantly in arm I, the salt bridge is formed with the side chain of Lys367, while Tyr327 also contacts the carboxylate group of the ligand. H3 residues Cys285 and Ser289 additionally participate in recognition in this binding mode. The ELE contribution is comparable to that of binding mode 1a.

##### 3.5.6.3 CBD

In all three modes, CBD is located predominantly at the center of the LBP (Figure 11). In mode 2b, one of the CBD hydroxyl groups interacts with the backbone of H3, involving residues Ile281 and Cys285; these contacts are maintained throughout the simulation, particularly with Cys285. Nevertheless, the pairwise RMSD plot displays high off-diagonal values, indicative of the sampling of multiple positions over the course of the simulation.

Mode 4 exhibits comparable stability; however, the initial interaction between one of the CBD hydroxyl groups and Phe363 is lost within a few nanoseconds after 500 ns? The side chain of Tyr327, while remaining in proximity to the ligand throughout the simulation, displays a broad distribution of relative orientations, reflecting the limited stability of this interaction.

In binding mode 5, following initial fluctuations, CBD stabilizes into a conformation in which it forms polar interactions with three LBP residues. One hydroxyl group interacts simultaneously with the backbone of Cys285 and the side chain of Ser289. The other hydroxyl forms a hydrogen bond with the side chain hydroxyl of Tyr327, with which an aromatic-aromatic interaction is also maintained. This three-point anchoring renders mode 5 more stable than mode 2b and yields a slightly more favorable ELE contribution.

##### 3.5.6.4 CBDA

All three binding modes analyzed for CBDA display good stability (Figure 12). Two of them are located entirely within arm I (modes 2c and 3), while the remaining one (mode 5) is positioned at the center of the LBP and partially in arm II. Mode 3 is the most stable throughout the simulation, with the cannabinoid anchored through persistent interactions involving all three of its polar groups: the carboxylate forms a salt bridge with Arg288, the adjacent hydroxyl forms a hydrogen bond with Ser289, and the other hydroxyl interacts with Tyr327 and His449. The tightly clustered distance and angle values with respect to Tyr327 are indicative of a highly conserved aromatic-aromatic interaction.

In mode 5, the cannabinoid is likewise anchored at three points, again involving Arg288. A network of polar interactions is observed: the guanidinium group contacts both the carboxylate and the adjacent hydroxyl, while Ser342 contributes by interacting with the carboxylate. On the opposite side, the other hydroxyl forms hydrogen bonds with the backbone atoms of H3 residues Ile281 and Cys285.

Mode 2c is slightly less stable, likely because the cannabinoid is held solely through its carboxylate group, which forms a highly persistent salt bridge with Lys367 and a hydrogen bond with Tyr327. The atoms involved remain in very close proximity throughout the simulation with virtually no fluctuations. Accordingly, the ELE contribution calculated for this mode is the most favorable among all modes and all ligands. The aromatic-aromatic interaction with Tyr327 is also conserved, although to a lesser extent than in mode 3.

## 4 Interpretation of results and comparison with previous studies

Determining the molecular mechanisms underlying cannabinoid action on PPARγ will be essential to deepen our understanding of the medicinal effects of cannabis and, in our view, may help guide more rational therapeutic strategies based on this traditional medicine. In particular, characterizing the binding modes of the most abundant cannabinoids represents a crucial first step toward understanding the therapeutic profile of each compound and of their combinations.

Cannabinoids interact with a broad range of proteins through a variety of mechanisms. For example, in CB receptors they bind within an internal cavity (Gloriam et al., 2024), whereas in Cys-loop channels they exploit small pockets formed at the surface of transmembrane helices (Alvarez and Alves, 2024). Given the dimension and the flexibility of the PPARγ LBP, together with the structural characteristics of cannabinoids, defining their binding mode is challenging and demands a comprehensive approach. Indeed, precedents exist in the NR family for ligands capable of adopting more than one binding conformation within the same receptor. For instance, SR12813 displays three distinct binding modes in the pregnane X receptor (PXR) (Watkins et al., 2001). Moreover, several crystallographic studies have shown that the PPARγ LBP can accommodate more than one ligand simultaneously. Itoh et al. reported a PPARγ structure in which the LBP was occupied by two hydroxylated fatty acids (Itoh et al., 2008), and both Waku et al. and Puhl et al. described structures in which two structurally distinct ligands co-crystallized within the cavity (Waku et al., 2010; Puhl et al., 2012). In addition, NMR analyses have demonstrated that two synthetic ligand molecules can bind simultaneously to PPARγ (Hughes et al., 2014). These findings suggest that cannabinoids may bind to PPARγ through multiple or alternative binding modes. Previous computational studies by D’Aniello et al. (2019) and Palomares et al. (2020) employed molecular docking or docking followed by MD simulations to investigate the binding modes of THCA and CBDA, respectively, showing that these cannabinoids can indeed adopt multiple binding orientations within the LBP. These studies yielded important initial insights into cannabinoid-PPARγ interactions, although they were based on a limited number of receptor structures and did not systematically explore the full spectrum of possible binding modes. In contrast, this work employed a large-scale docking strategy across more than 70 PPARγ structures, followed by an exhaustive MD-based evaluation of binding mode stability, persistent ligand-receptor interactions, and comparative energetic analyses of all four major cannabinoids, resulting in a cumulative simulation time of 36 μs.

Docking was carried out using two strategies: against the unliganded receptor crystal structure on the one hand, and against a broad set of structures co-crystallized with diverse ligands on the other, in order to exploit the conformational variability of the LBP. For THC and THCA, the predominant docking solutions were consistent across both strategies, positioning the cannabinoid at the center of the LBP. These solutions subsequently exhibited good stability in MD trajectories (modes 1a of THC and THCA), suggesting that they may indeed represent viable binding modes. Beyond these results, however, other alternative docking solutions also led to stable binding modes in the simulations, with comparable energetic contributions. A mode located in arm I was identified for THC, characterized by the formation of an aromatic-aromatic interaction between the resorcinol ring and the side chain of an aromatic residue (mode 4). THCA was also able to adopt a binding mode in arm I, establishing several polar interactions with LBP residues (mode 5b). These modes may therefore also play a relevant role in the modulation of PPARγ activity by these cannabinoids. Notably, binding modes 4 and 5b exhibit features similar to those reported previously (Palomares et al., 2020), with THCA contacting Ser342 or Ser289, respectively.

For CBD and CBDA, no agreement was observed between the solutions obtained for PPARγ-apo and those obtained from the ensemble of holo structures. Furthermore, in contrast to THC and THCA, the latter strategy did not yield a markedly predominant solution; instead, several solutions of similar frequency were detected upon clustering of the poses. Nevertheless, the selection and analysis of representative solutions allowed the identification of binding modes with acceptable stability in MD simulations. A binding mode was characterized in which CBDA is located at the intersection of arms II and III, anchored by multiple interactions through all polar groups of the cannabinoid (mode 5). Remarkably, by using MD simulations, D'Aniello et al. identified two possible binding modes, one of which closely resembles our mode 5 (D’Aniello et al., 2019). Notably, our analysis identified an additional binding mode within arm I, again stabilized by multiple polar ligand-receptor interactions that maintain the cannabinoid in a very stable bound conformation (mode 3). The stability of CBD binding modes was lower than that of the other cannabinoids, likely due to the greater flexibility of its scaffold and the absence of an acidic group. Nevertheless, a binding mode at the intersection of arms II and III could be identified, in which CBD maintains persistent polar and aromatic-aromatic interactions with the receptor (mode 5). In this way, at least one stable binding mode was identified for each cannabinoid.

Experimental studies have established a relative order of affinity and activity for these cannabinoids at PPARγ (THCA > CBDA >> THC > CBD), as determined by both binding and transcriptional activation assays (Nadal et al., 2017). In a PPARγ competitor assay, Ki values of 209 nM, 5,548 nM, 7,630 nM, and >11,660 nM were obtained for THCA, THC, CBDA, and CBD, respectively. Consistently, transcriptional activation assays showed that THCA induced significant activation at 1 μM, whereas THC required concentrations of 25 μM to achieve a comparable effect; similarly, CBDA displayed significant activation at 10 μM, while CBD elicited significant activation only at 25 μM. Together, these findings support the notion that acidic cannabinoids exhibit greater potency toward PPARγ than the corresponding decarboxylated forms. Although our analysis, based on the number of binding modes, their stability, and the persistence of polar and aromatic-aromatic interactions, does not fully account for why THCA is a better ligand than CBDA in those experiments, it does explain the large difference between neutral cannabinoids and their acidic forms, whose binding modes are favored by the formation of strong receptor-ligand salt bridges. Furthermore, the lower activity of CBD observed experimentally is consistent with the comparatively less stable binding modes identified in our simulations.

## 5 Limitations of the study

The computational methodology here employed has some limitations that should be acknowledged. First, docking outcomes depend on the choice of receptor conformations and the scoring function employed, both of which are unable to fully account for the energetics of ligand-receptor interactions. Moreover, although MD simulations were extended to the microsecond timescale, this does not guarantee that the conformational space of the highly flexible LPB structure has been exhaustively sampled. Finally, MM/GBSA free energy calculations are associated with inherent uncertainties and should be regarded as relative estimates of binding affinity rather than absolute values.

## 6 Conclusion and future prospects

This work provides a foundation for future studies addressing the molecular basis of cannabinoid action on PPARγ. For example, targeted mutagenesis studies could be designed to evaluate the role of the basic residues Arg280, Arg288, and Lys367 in the recognition of THCA and CBDA, or the role of Ser342 in the binding of all four cannabinoids. Moreover, these findings could serve as a starting point to explore the allosteric mechanism by which these small lipophilic molecules influence receptor structure and dynamics. Finally, the strategy described here may also be applied to determine the binding modes of these natural compounds in other members of the nuclear receptor family, including PPARα and PPARδ, as well as to investigate the binding modes of minor cannabinoids, such as cannabichromene and cannabigerol.

## Data Availability

The original contributions presented in the study are included in the article/Supplementary Material, further inquiries can be directed to the corresponding author.
