# Supplementary material for: Multiple binding modes underlie Cannabis sativa cannabinoids recognition by peroxisome proliferator-activated receptor gamma
Source: Front Bioinform. 2026 Aug 10;6:1893303. doi: 10.3389/fbinf.2026.1893303 (PMC13500514; doi:10.3389/fbinf.2026.1893303)
Supplement: Supplementary file 1 [file DataSheet1.pdf]

frontiers

Supplementary Material

Multiple binding modes underlie cannabinoid recognition by peroxisome proliferator-activated receptor gamma

N. R. Carina Alves[1,2], Justina Miranda[1] and Lautaro D. Alvarez[1,2*]

[1]Departamento de Química Biológica, Facultad de Ciencias Exactas y Naturales, Universidad de Buenos Aires, Ciudad Universitaria, Buenos Aires C1428EGA, Argentina

[2]UMYMFOR, CONICET - Universidad de Buenos Aires, Ciudad Universitaria, Buenos Aires C1428EGA, Argentina

1 Supplementary Figures ………………………………………………2-18

2 Supplementary Tables …...……………………………………........19-25

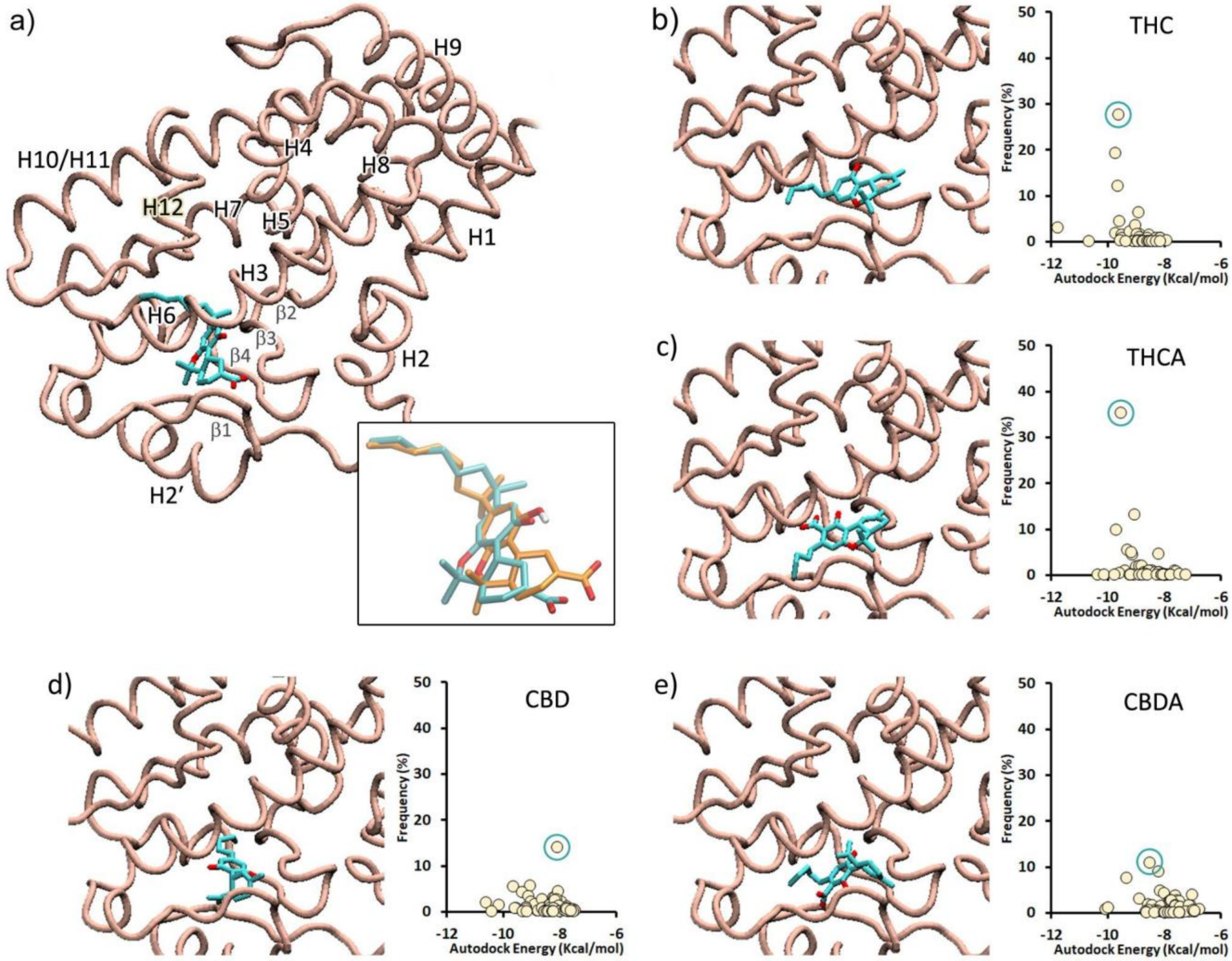

Supplementary Figure S1. (a) Crystal structure of the PPARγ LBD/AJA complex (PDB ID: 2OM9). Secondary structure elements are labeled. The inset shows the superposition of the crystallographic conformation (cyan carbon atoms) and the top-ranked redocked pose (orange carbon atoms) of AJA. The predicted binding energy was −9.7 Kcal/mol, and the corresponding docking cluster accounted for 67% of the generated poses. (b-e) Docking of cannabinoids into the PPARγ LBD/AJA structure (PDB ID: 2OM9). Left: lowest-energy solution from the most frequent cluster; right: Autodock4 frequencies and energies for THC (b), THCA (c), CBD (d), and CBDA (e).

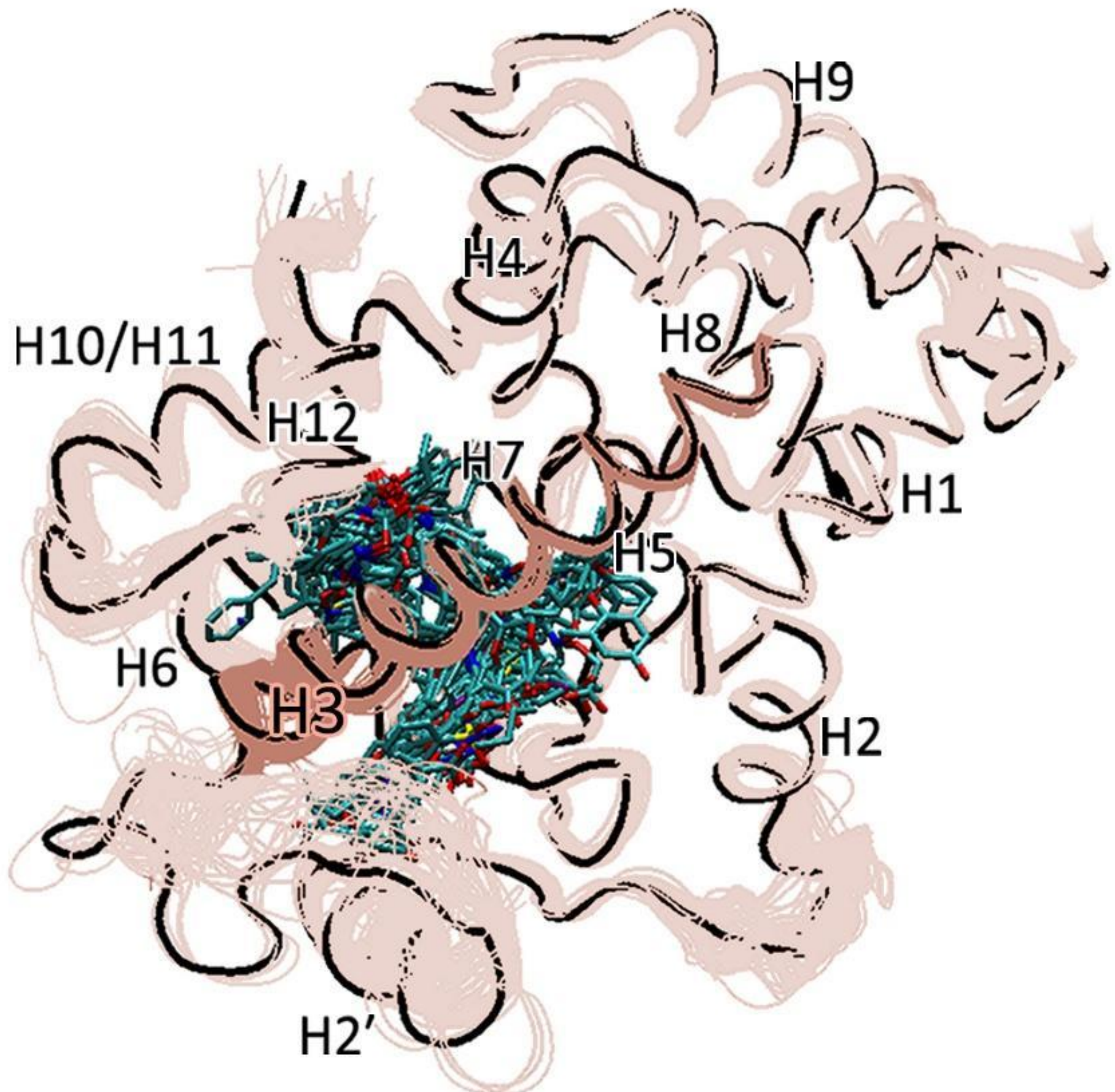

Supplementary Figure S2. Superposition of 70 PPARγ LBD-holo structures, showing the co-crystallized ligand in each case. The backbone of the PPARγ LBD-apo structure (PDB ID: 1PRG) is shown in black for reference.

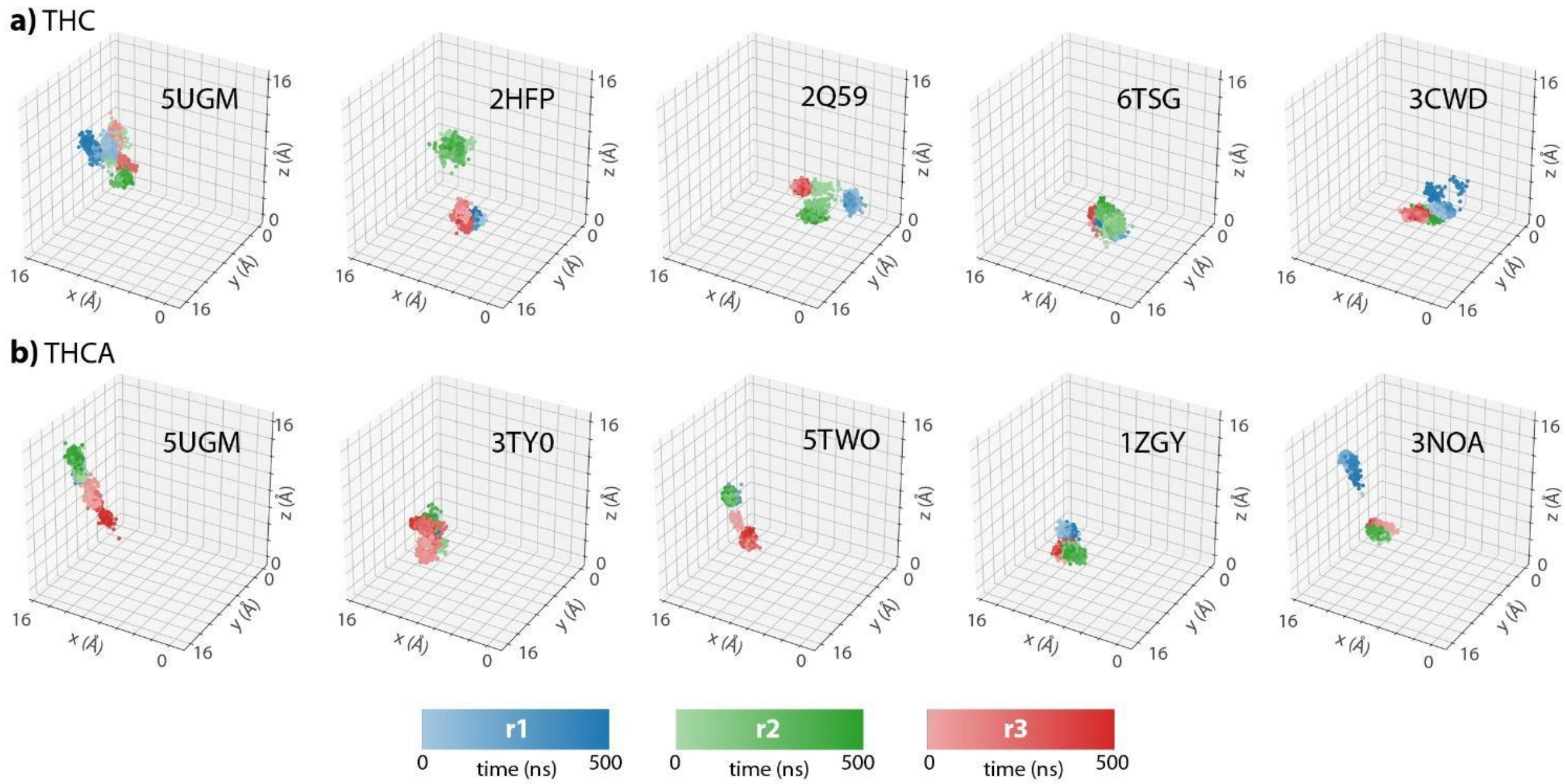

Supplementary Figure S3. Position of the ligand center of mass throughout the MD trajectories of PPARγ/THC (a) and PPARγ/THCA (b) complexes.

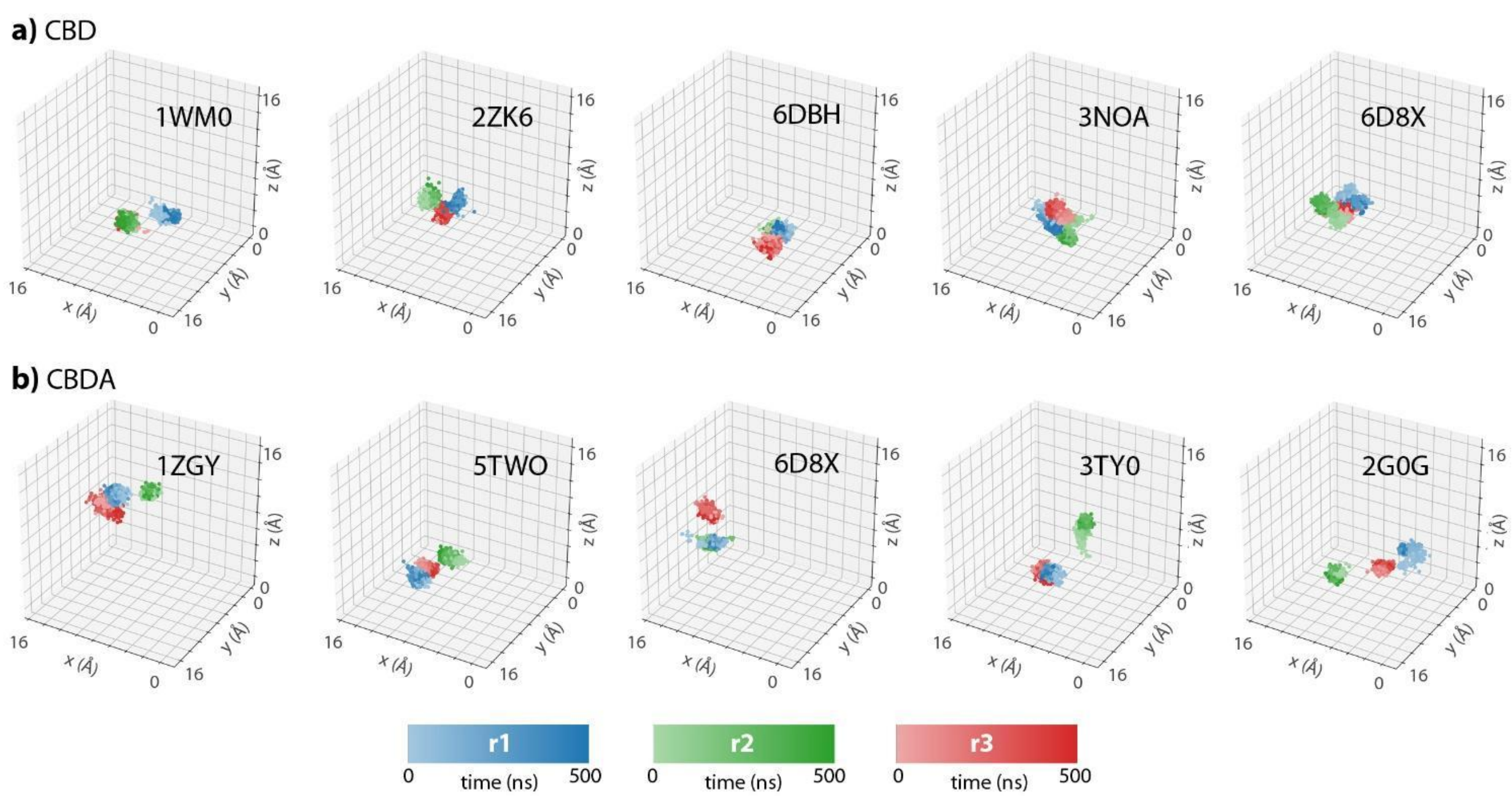

Supplementary Figure S4. Position of the ligand center of mass throughout the MD trajectories of PPARγ/CBD (a) and PPARγ/CBDA (b) complexes.

THC

	5UGM			2HFP			2Q59			6TSG			3CWD		
	r1	r2	r3	r1	r2	r3	r1	r2	r3	r1	r2	r3	r1	r2	r3
PCA variance	0.73	0.83	1.10	0.08	0.24	0.92	0.10	0.97	0.07	0.45	0.89	0.23	2.54	0.27	0.17
# Polar Interacions	1	0	1	0	0	1	2	1	2	1	0	0	0	0	0
# Aromatic Interactions	0	0	0	1	0	0	2	1	2	0	0	0	0	0	0

THCA

	5UGM			3TYO			5TWO			1ZGY			3NOA		
	r1	r2	r3	r1	r2	r3	r1	r2	r3	r1	r2	r3	r1	r2	r3
PCA variance	0.22	0.31	1.03	0.10	1.31	0.82	0.12	0.20	0.66	0.14	0.24	0.12	0.20	0.12	0.23
# Polar Interacions	5	5	0	0	0	0	2	2	5	4	0	0	6	5	3
# Aromatic Interactions	0	0	0	0	0	0	0	0	0	0	0	0	0	0	0

CBD

	1WM0			2ZK6			6DBH			3NOA			6D8X		
	r1	r2	r3	r1	r2	r3	r1	r2	r3	r1	r2	r3	r1	r2	r3
PCA variance	0.62	0.18	0.18	0.11	0.42	0.20	0.17	0.10	0.36	0.45	0.74	0.60	0.52	0.78	0.44
# Polar Interacions	0	0	2	0	2	1	0	0	0	1	0	0	3	0	2
# Aromatic Interactions	0	0	0	0	0	0	0	0	0	2	0	0	1	0	0

CBDA

	1ZGY			5TWO			6D8X			3TY0			2G0G		
	r1	r2	r3	r1	r2	r3	r1	r2	r3	r1	r2	r3	r1	r2	r3
PCA variance	0.17	0.08	0.28	0.13	0.23	0.27	0.17	0.30	0.23	0.10	0.32	0.12	0.28	0.19	0.52
# Polar Interacions	3	1	2	0	1	2	5	3	0	3	7	3	4	3	7
# Aromatic Interactions	0	1	0	0	0	0	1	1	0	0	0	0	2	1	0

Supplementary Figure S5. Variance of the PCA (PC1/PC2) projection, number of polar interactions, and number of aromatic-aromatic interactions with an occupancy greater than 40% along the simulation, for each of the simulated systems.

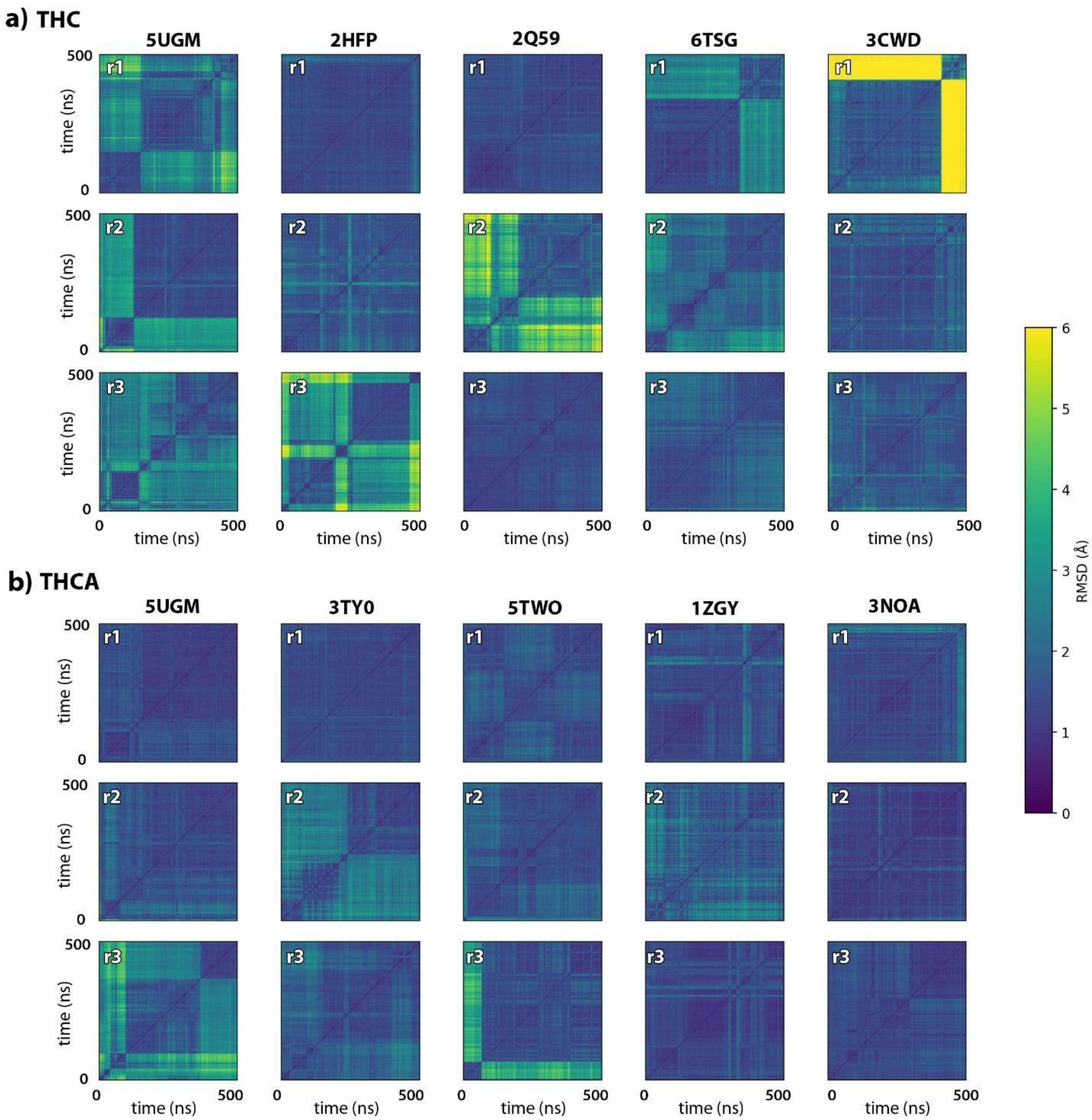

Supplementary Figure S6. Pairwise ligand RMSD computed for the THC (a) and THCA (b) systems.

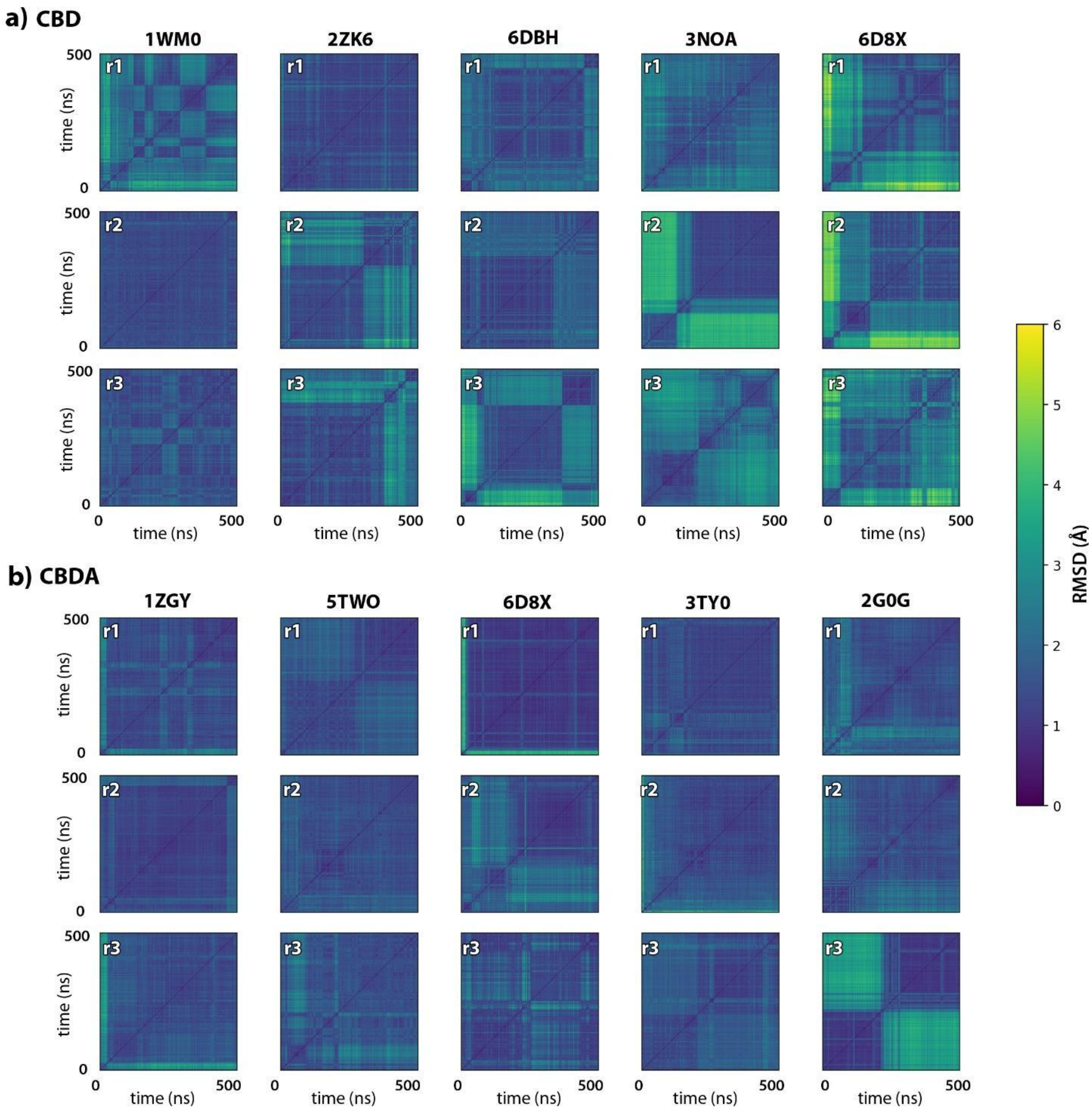

Supplementary Figure S7. Pairwise ligand RMSD computed for the CBD (a) and CBDA (b) systems.

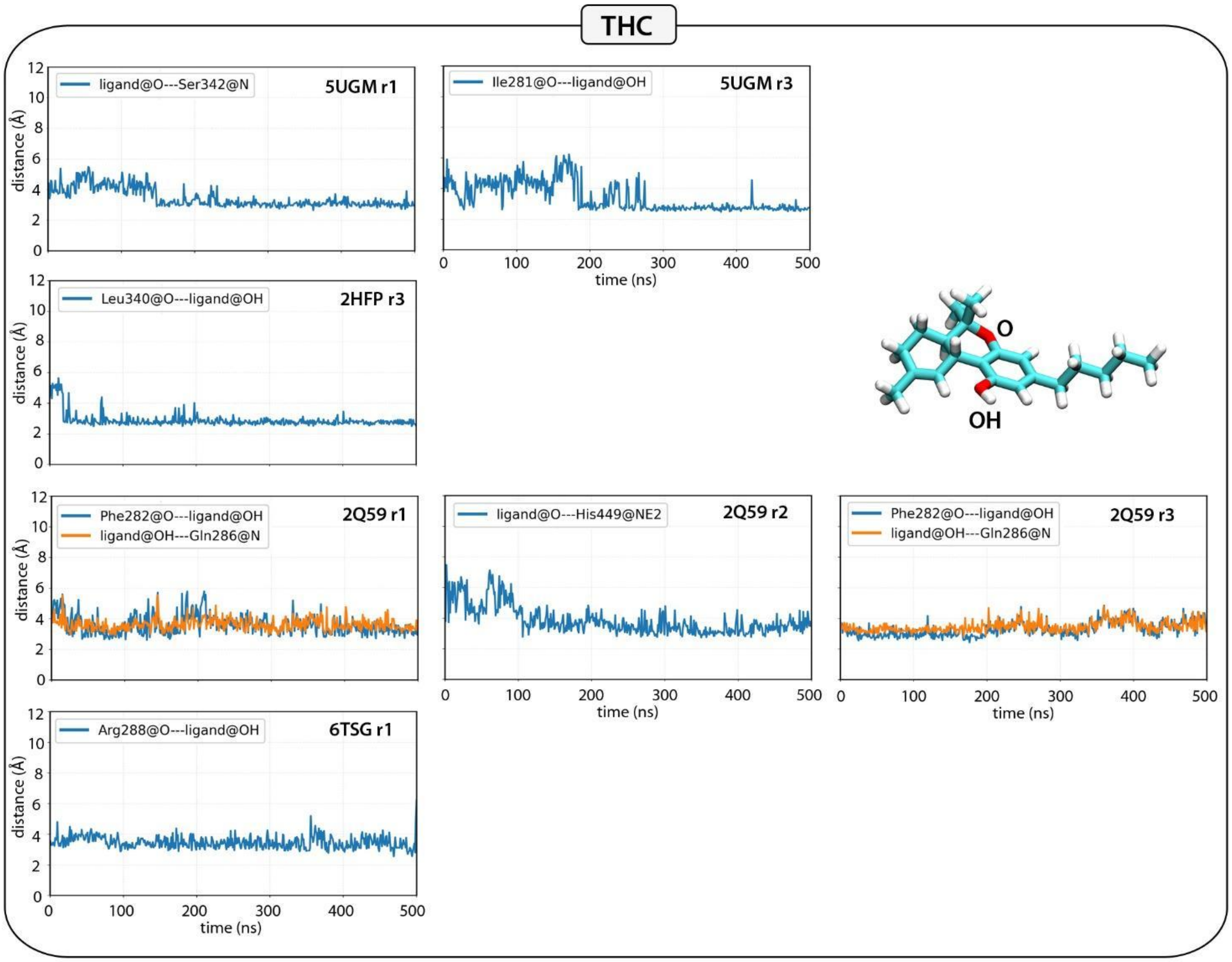

Supplementary Figure S8. Time evolution of the distances between PPARγ and THC atoms involved in the polar interaction identified along the simulations.

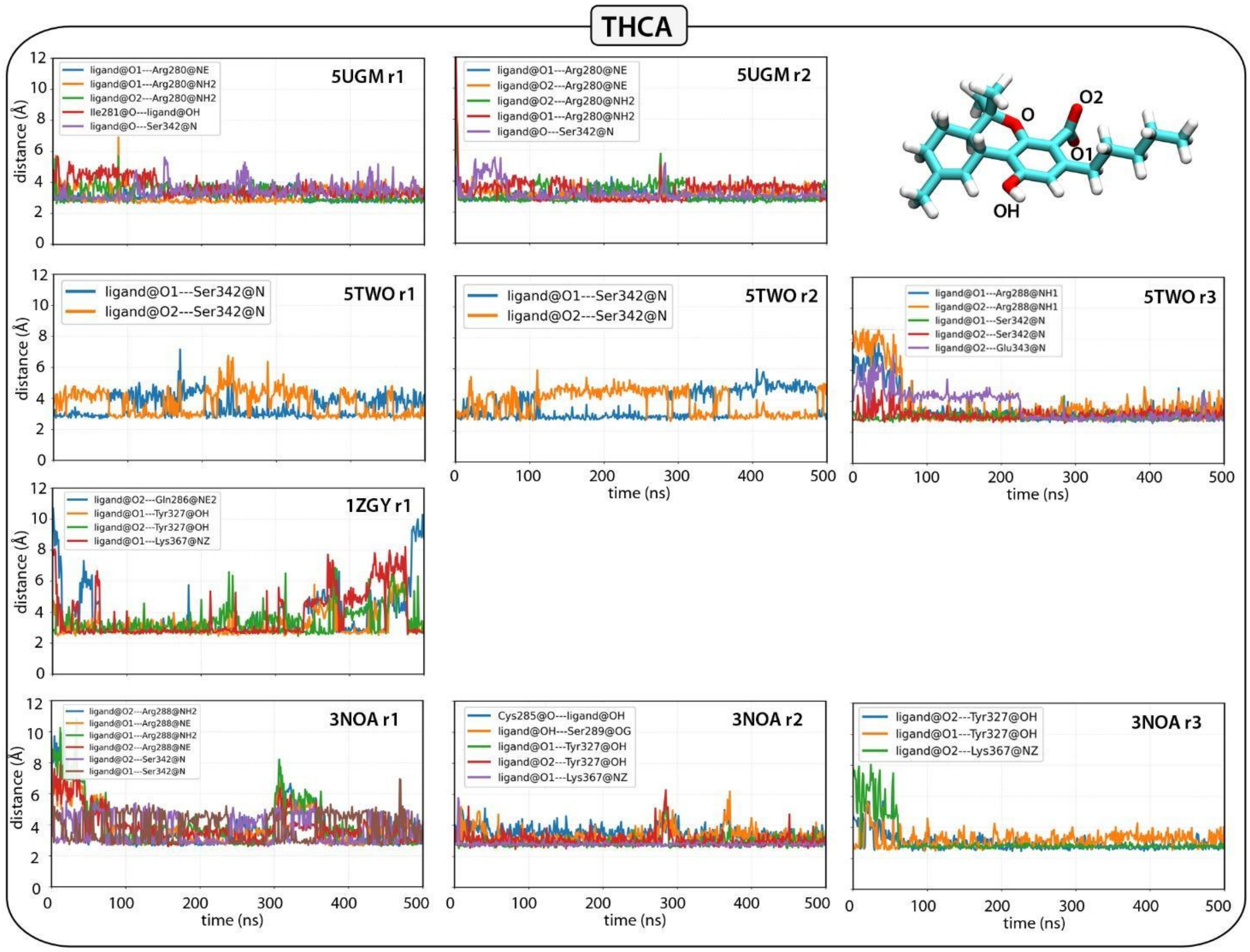

Supplementary Figure S9. Time evolution of the distances between PPARγ and THCA atoms involved in the polar interaction identified along the simulations.

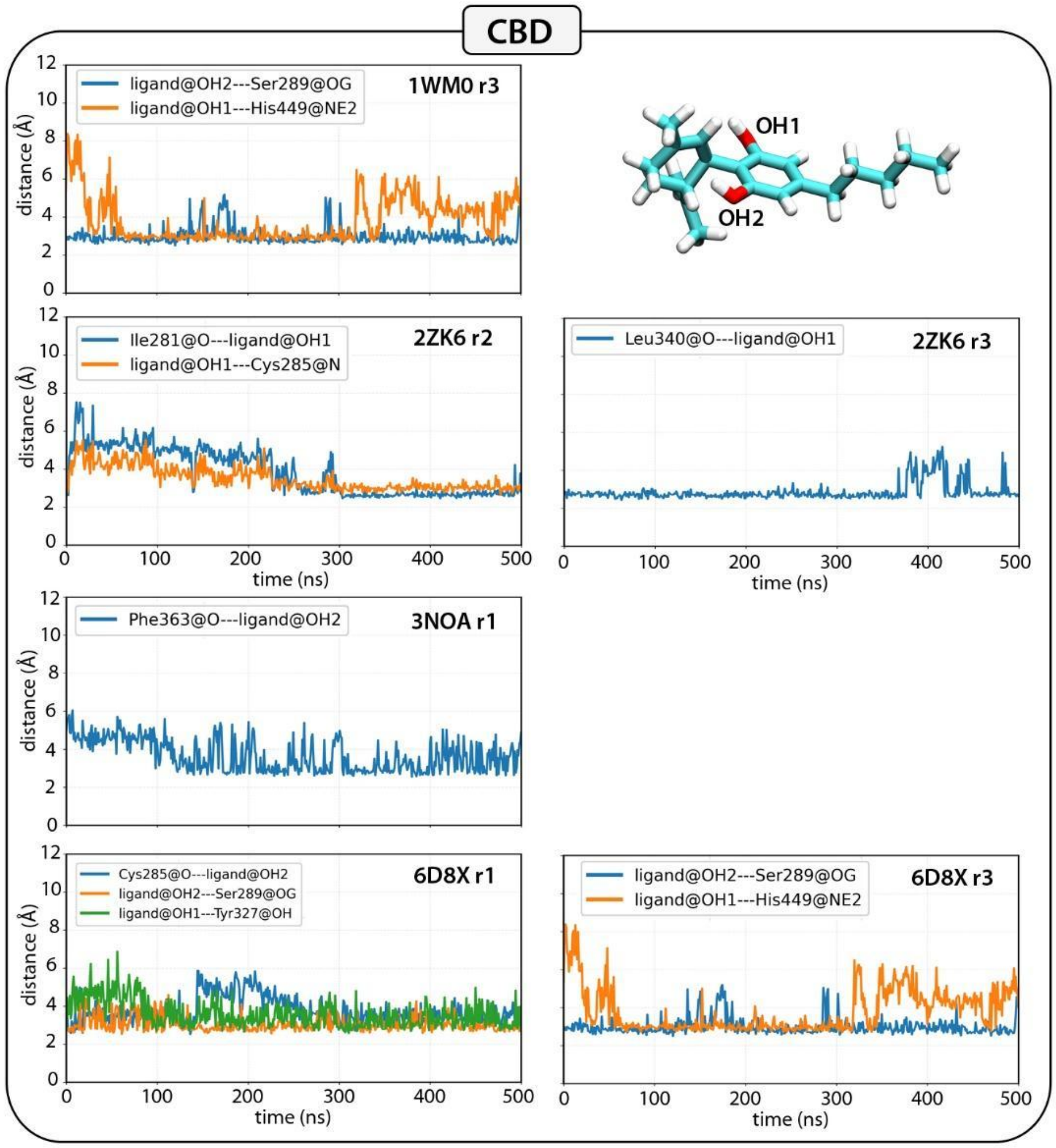

Supplementary Figure S10. Time evolution of the distances between PPARγ and CBD atoms involved in the polar interaction identified along the simulations.

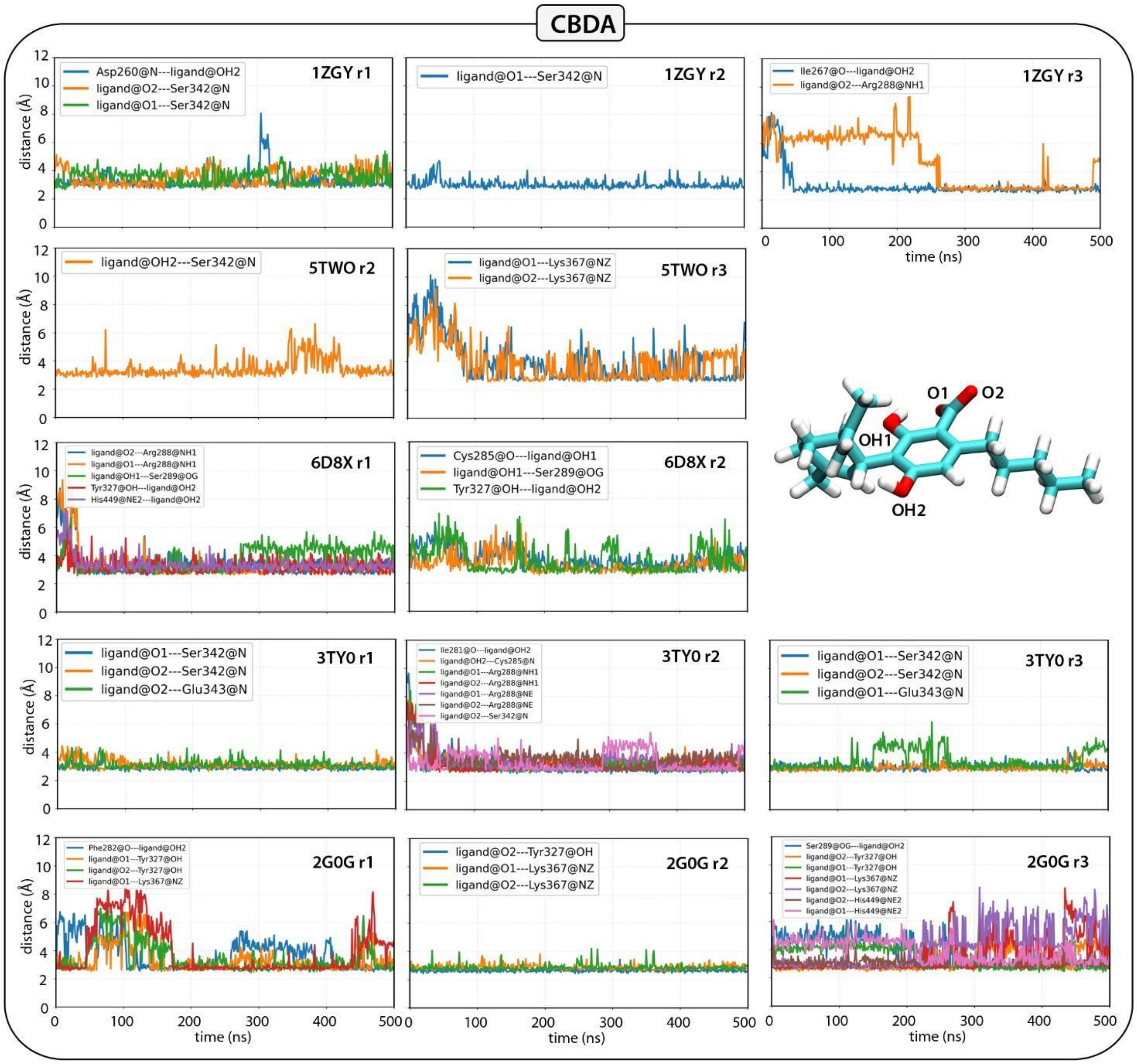

Supplementary Figure S11. Time evolution of the distances between PPARγ and CBDA atoms involved in the polar interaction identified along the simulations.

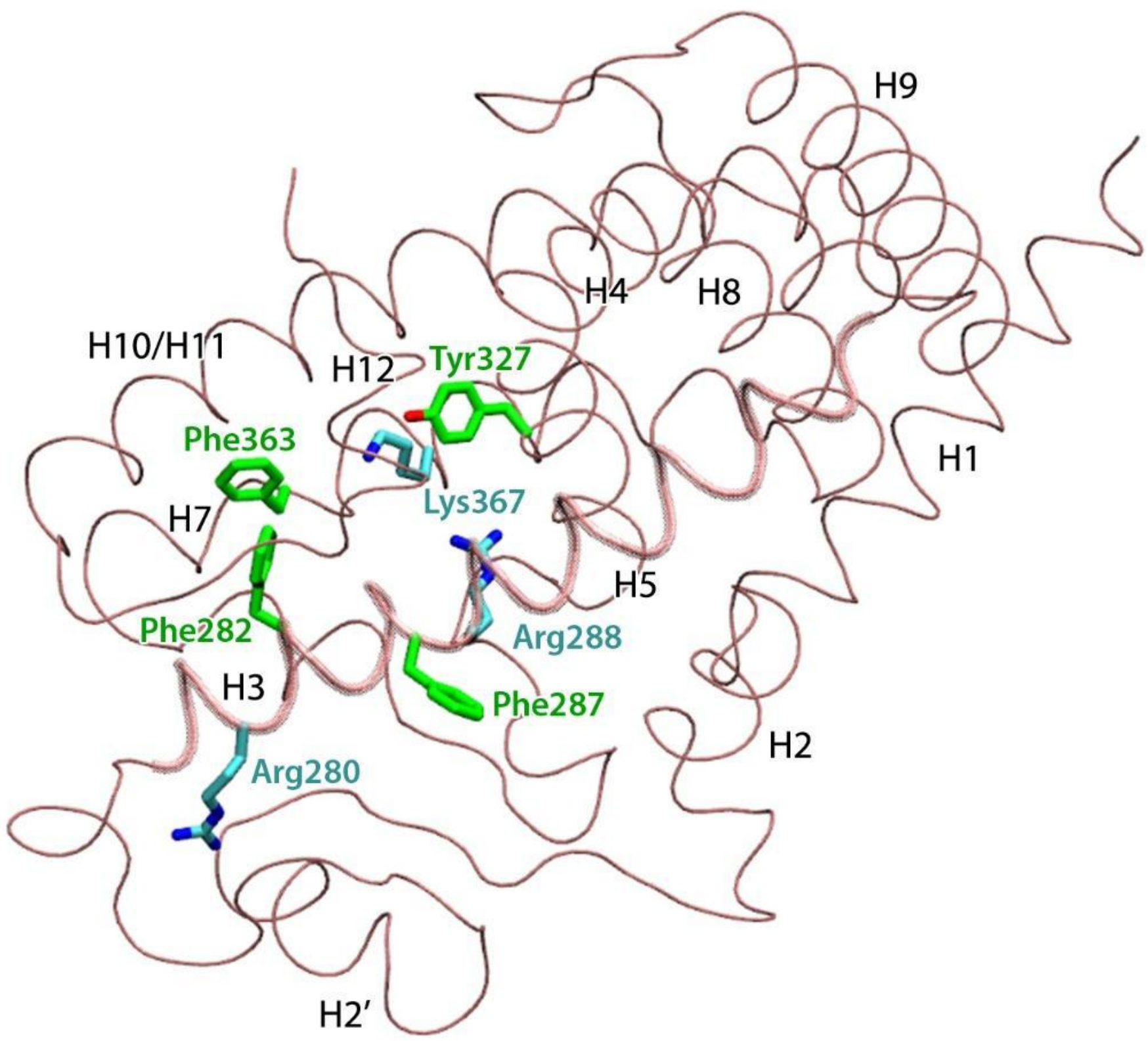

Supplementary Figure S12. Location of the basic residues (cyan carbons) and aromatic residues (green carbons) that interact with the cannabinoids in at least one of the MD simulations, shown on the PPARγ LBD-apo structure (PDB ID: 1PRG).

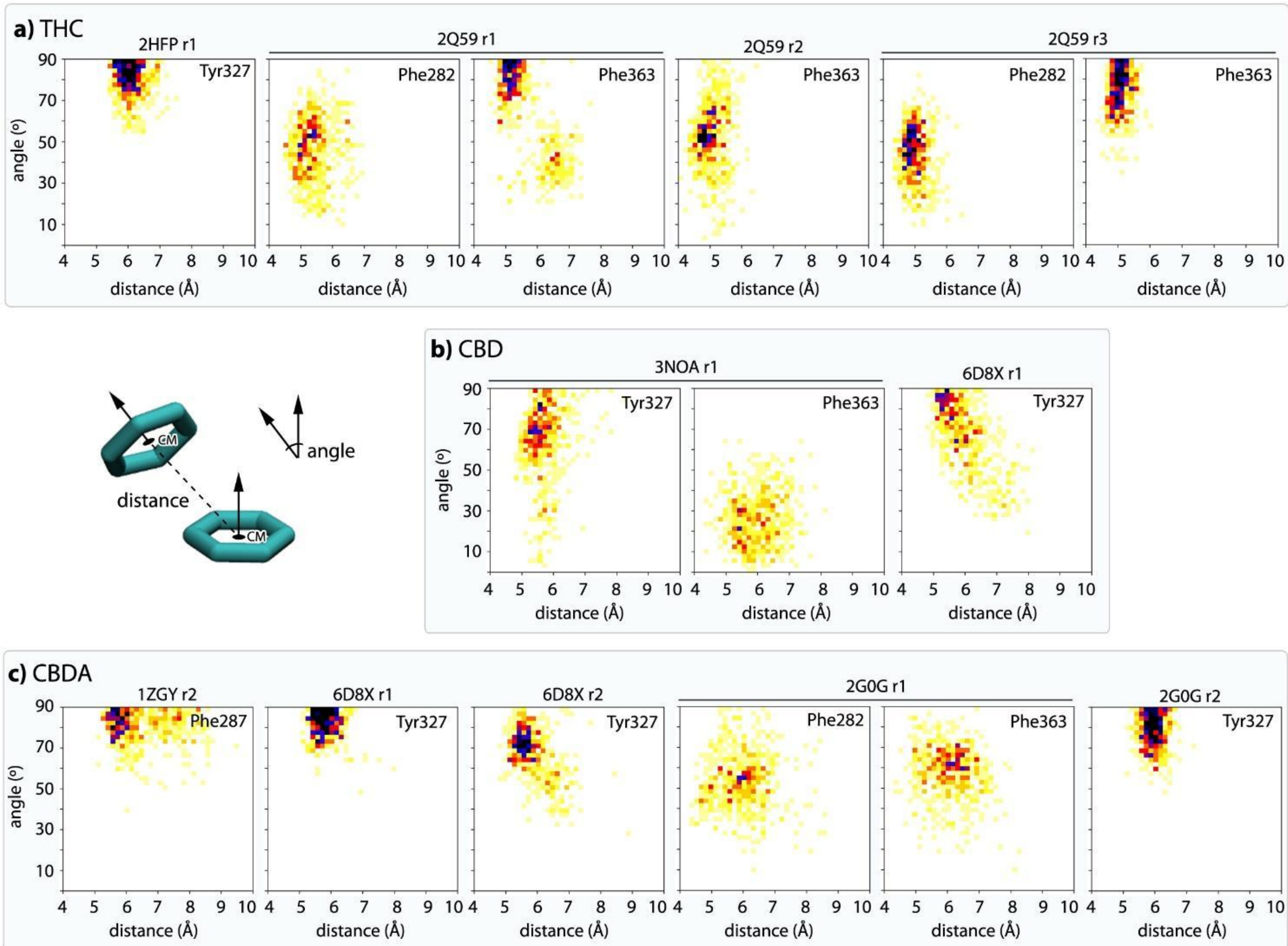

Supplementary Figure S13. 2D histograms showing the distribution of the distance between the centers of mass and the angle between the normal vectors of the cannabinoid resorcinol ring and the side chains of the indicated aromatic residues.

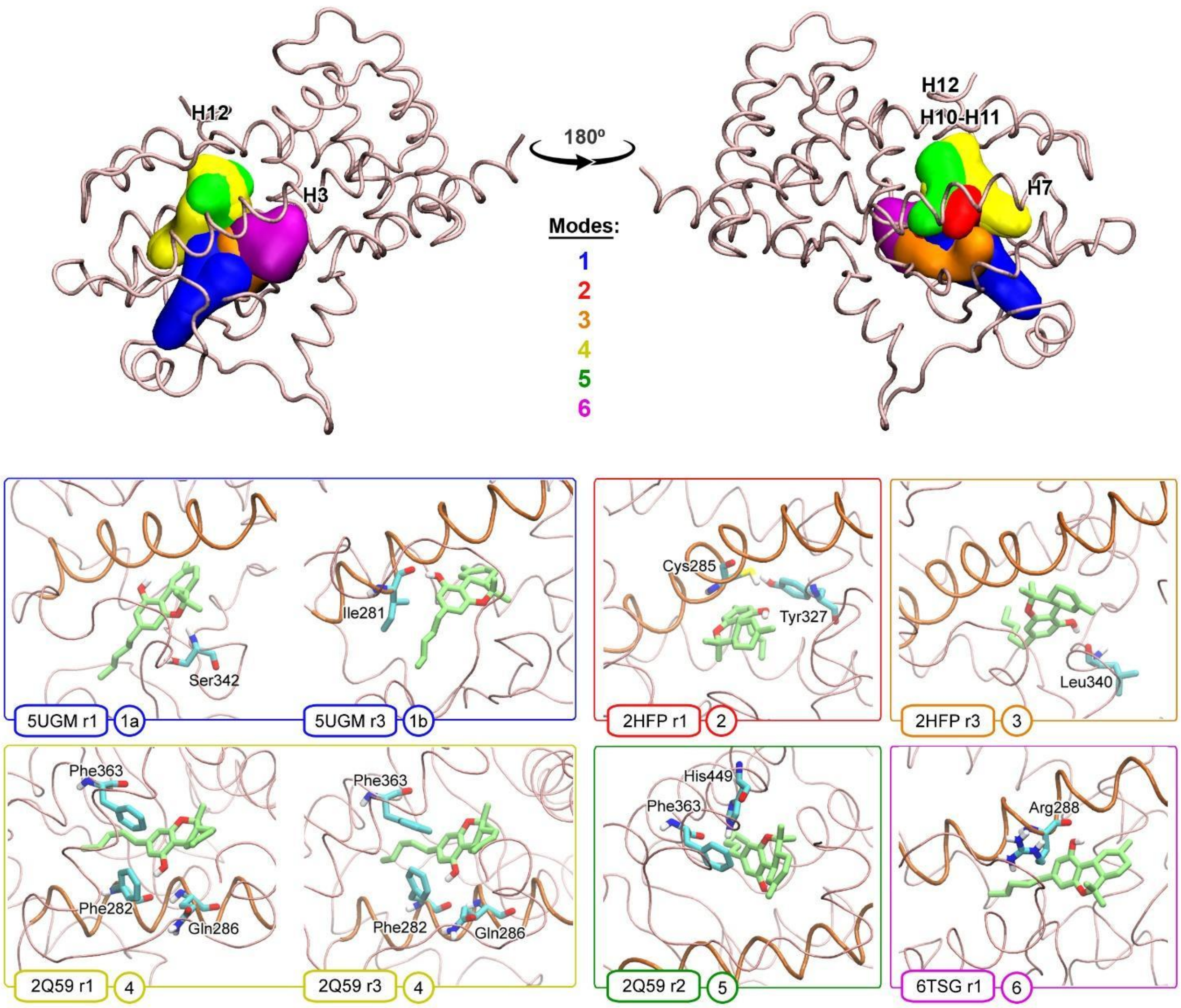

Supplementary Figure S14. Binding modes obtained for THC. Representative snapshots of each principal cluster of different MD simulations are shown. THC (carbons depicted in lime), contacting residues (carbons depicted in cyan), helix 3 is highlighted. PDB ID, replica, and number of binding mode are indicated for each snapshot. Similar binding modes are grouped together.

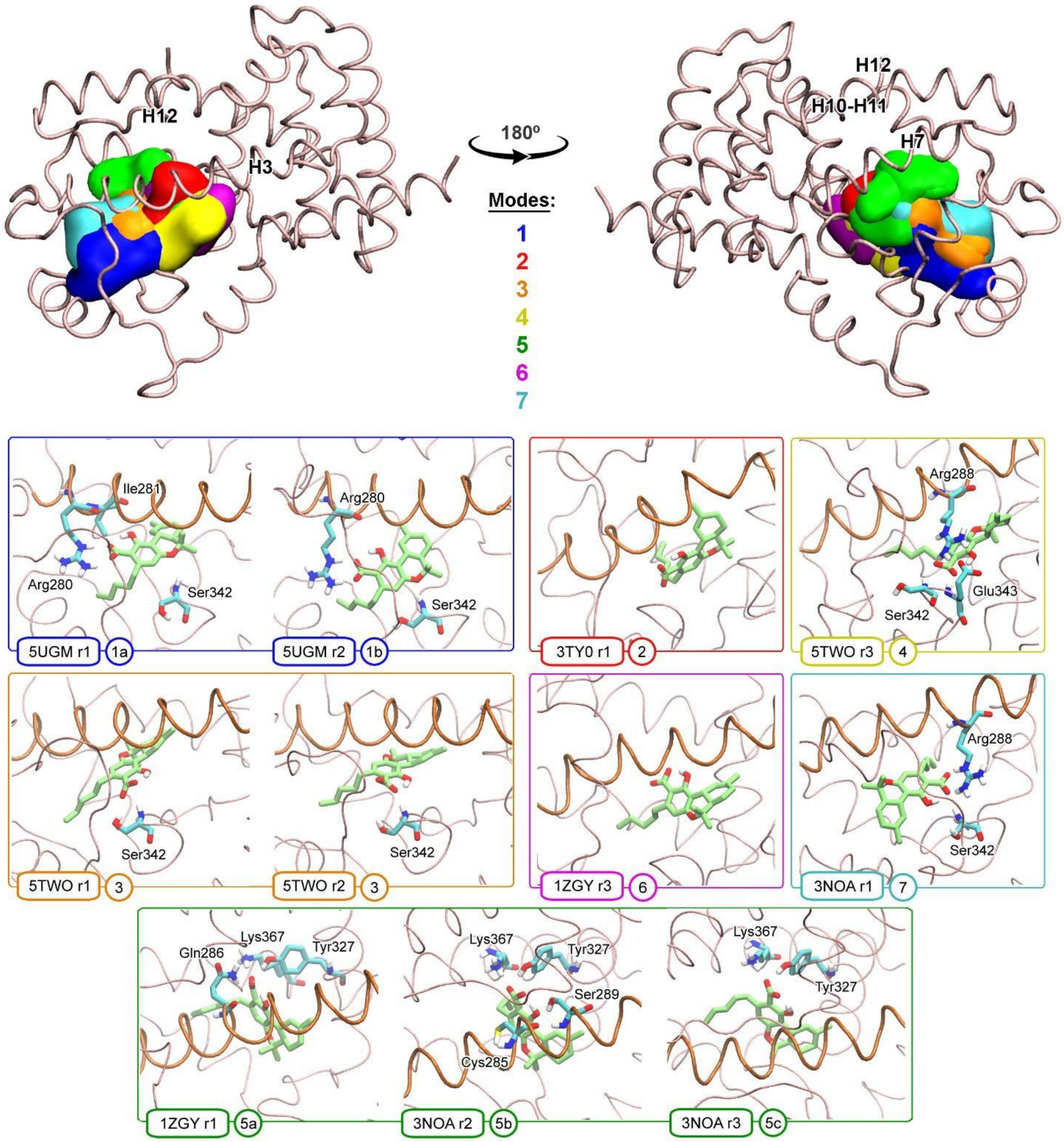

Supplementary Figure S15. Binding modes obtained for THCA. Representative snapshots of each principal cluster of different MD simulations are shown. THCA (carbons depicted in lime), contacting residues (carbons depicted in cyan), helix 3 is highlighted. PDB ID, replica, and number of binding mode for THCA are indicated for each snapshot. Similar binding modes are grouped together.

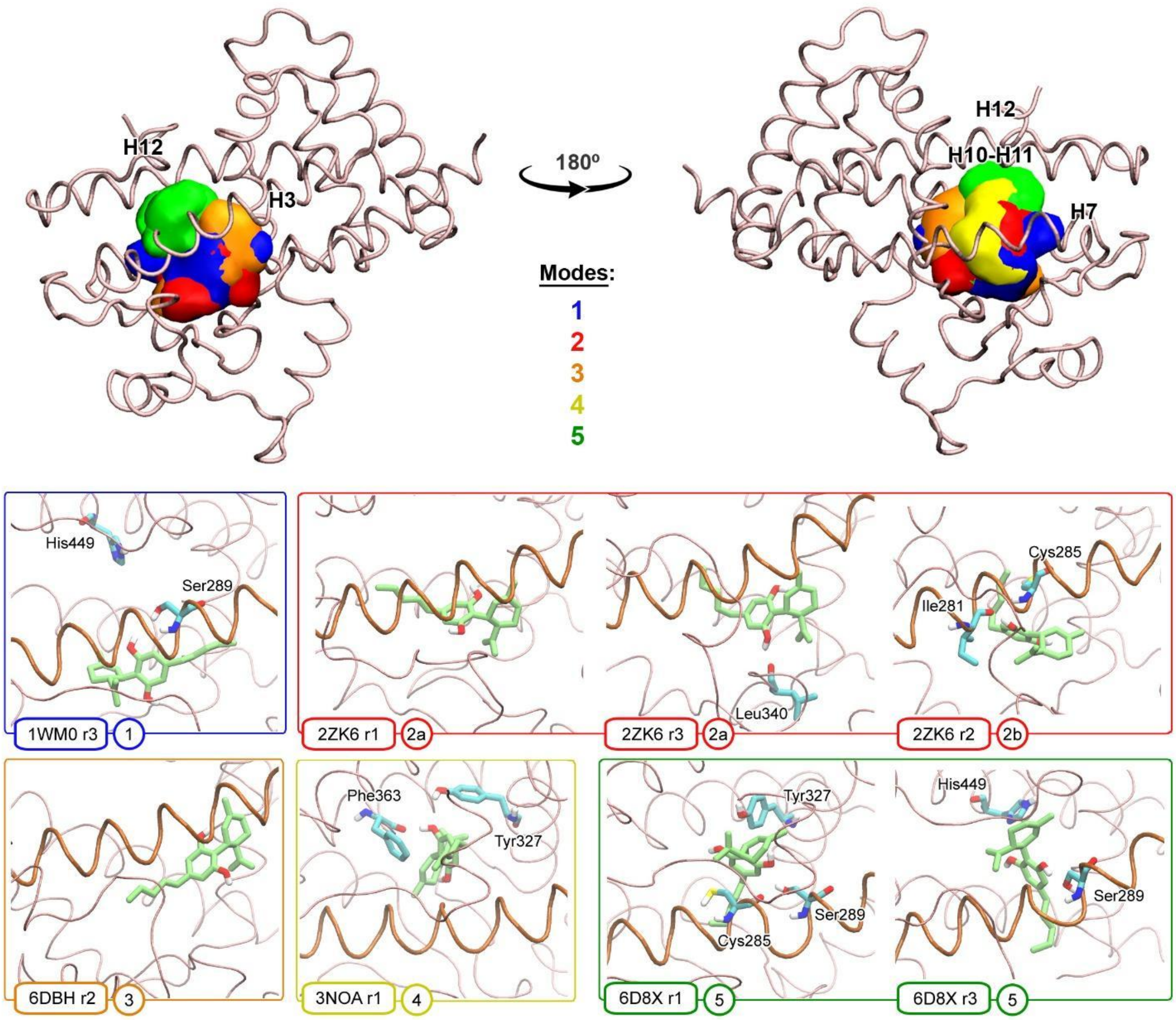

Supplementary Figure S16. Binding modes obtained for CBD. Representative snapshots of each principal cluster of different MD simulations are shown. CBD (carbons depicted in lime), contacting residues (carbons depicted in cyan), helix 3 is highlighted. PDB ID, replica, and number of binding mode for CBD are indicated for each snapshot. Similar binding modes are grouped together.

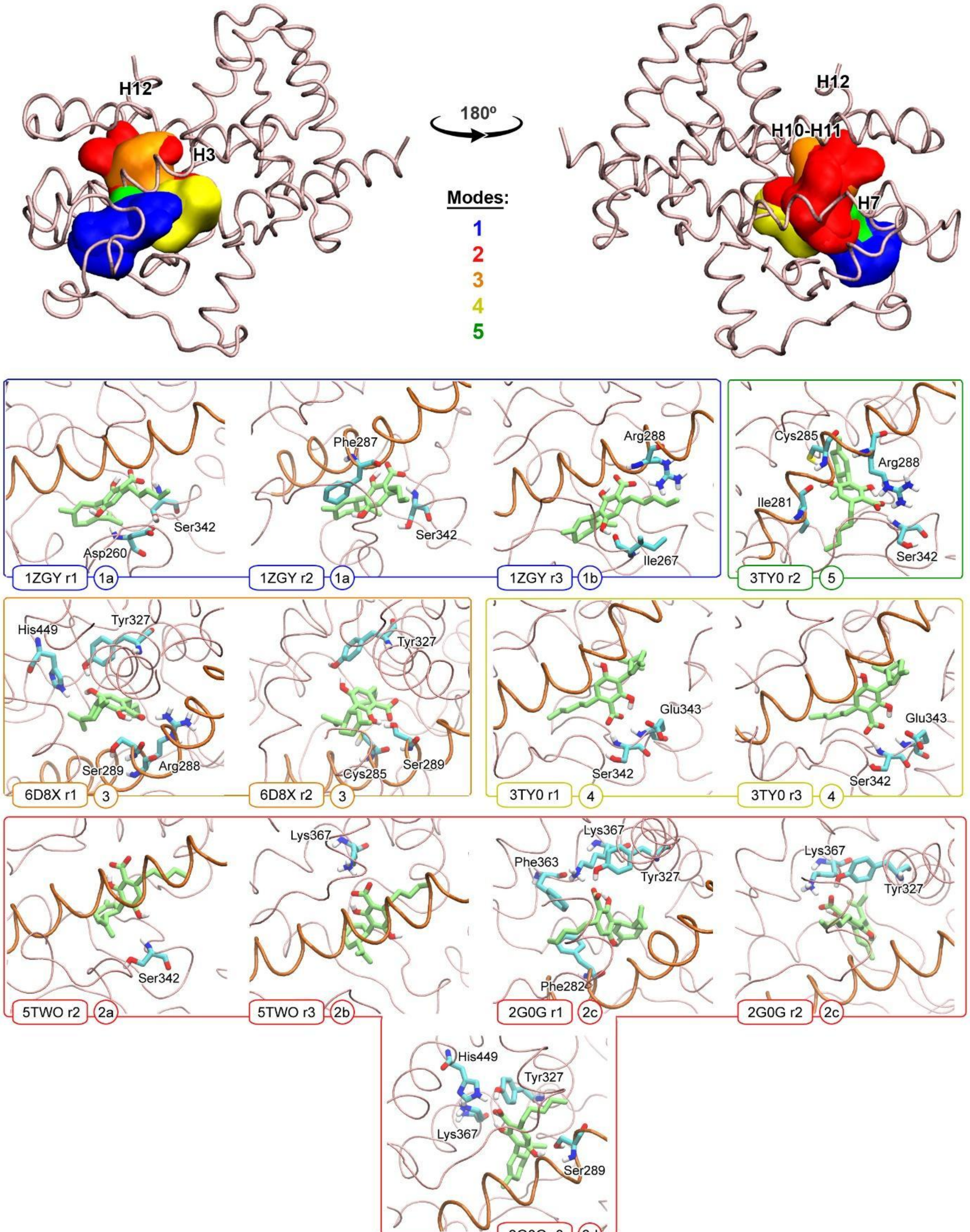

Supplementary Figure S17. Binding modes obtained for CBDA. Representative snapshots of each principal cluster of different MD simulations are shown. CBDA (carbons depicted in lime), contacting residues (carbons depicted in cyan), helix 3 is highlighted. PDB ID, replica, and number

of binding mode for CBDA are indicated for each snapshot. Similar binding modes are grouped together.

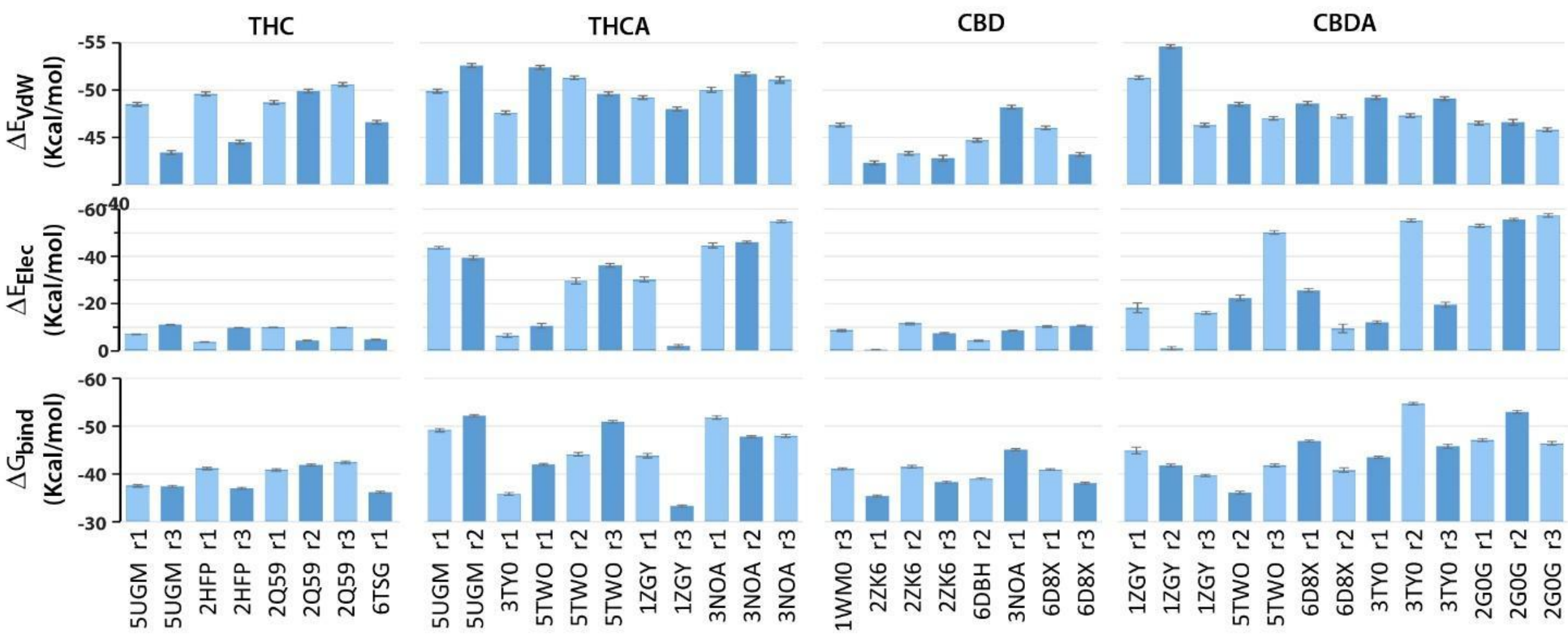

Supplementary Figure S18. MM/GBSA energy contributions for the selected systems.

PDB ID	Chain	Res (Å)	Missing residues	PDB ID	Chain	Res (Å)	Missing residues
1FM9	B	2.1	-	3TY0	A	2.0	-
1I7I	A	2.4	267 to 275	3VJH	A	2.2	262 to 274
1NYX	A	2.7	262 to 276	3VN2	A	2.2	263 to 272
1WM0	A	2.9	268 to 273	3VSO	A	2.0	265 to 273
1ZEO	A	2.5	264 to 271	4A4W	A	2.0	262 to 273
1ZGY	A	1.8	-	4FGY	A	2.8	290 to 294
2ATH	A	2.3	-	4HEE	A	2.5	241 to 243; 262 to 275
2FVJ	A	2.0	262 to 274	4JAZ	A	2.9	-
2G0G	A	2.5	-	4PRG	A	2.9	-
2HFP	A	2.0	-	4PVU	A	2.6	265 to 275
2I4Z	A	2.3	-	4R06	A	2.2	239 to 240; 260 to 275
2OM9	A	2.8	-	5GTN	A	1.9	269 to 273
2P4Y	A	2.3	229 to 223; 255 to 278	5TWO	A	1.9	-
2POB	A	2.3	241 to 243; 264 to 274	5UGM	A	2.1	-
2Q59	A	2.2	261 to 274; 285	6AD9	A	2.2	242; 262 to 274
2ZK5	A	2.5	-	6AUG	A	2.7	264 to 275
2ZK6	A	2.4	-	6AVI	A	2.3	263 to 274
2ZNO	A	2.4	263 to 270	6D3E	A	2.4	263 to 272
3ADS	A	2.3	264 to 275	6D8X	A	1.9	-
3BC5	A	2.3	242 to 244; 267 to 274	6DBH	A	2.6	-
3CWD	A	2.4	261 to 273	6DCU	A	3.0	263 to 272
3ET3	A	2.0	263 to 274	6DGO	A	3.1	267 to 274
3FUR	A	2.3	259 to 274	6ENQ	A	2.2	259 to 274
3G9E	A	2.3	262 to 274	6IJS	A	2.2	264 to 274
3H0A	B	2.1	244 to 261	6IZN	A	1.8	270 to 275
3HO0	A	2.6	-	6JF0	A	3.4	241; 264 to 274
3IA6	A	2.3	262 to 275	6K0T	A	1.8	263 to 274; 285
3NOA	A	2.0	-	6L89	A	2.1	264 to 274
3OSW	A	2.6	263 to 273	6MD0	A	2.0	240 to 241; 264 to 274
3QT0	A	2.5	241 to 244	6MS7	A	1.4	265 to 271
3R5N	A	2.0	242 to 243; 265 to 273	6TDC	A	2.3	289 to 302
3R8A	A	2.4	241 to 242; 263 to 273	6TSG	A	3.0	261 to 275
3R8I	A	2.3	-	6Y3U	A	2.6	261 to 275
3S9S	A	2.6	240 to 243; 269 to 274	6ZLY	A	1.8	260 to 275
3T03	A	2.1	296 to 298	7AWD	A	1.9	265 to 268

Supplementary Table S1. PPARγ crystal structures used for cannabinoid docking calculations.

THC				
**PDB ID**	**Lowest binding energy (Kcal/mol)**	**Mean binding energy (Kcal/mol)**	**Frequency (%)**	**Cluster**
3IA6	−10.5	−9.9	63.8	1
5UGM	−11.6	−10.9	60.7	1
1FM9	−11.0	−10.2	58.6	1
3ET3	−11.1	−10.3	53.0	1
2POB	−10.9	−10.3	51.7	1
6AUG	−11.3	−10.7	49.0	1
3BC5	−10.8	−10.3	46.4	1
3VSO	−10.2	−9.7	43.4	1
3S9S	−11.5	−10.6	39.8	1
1WM0	−13.7	−12.9	38.1	1
4JAZ	−11.1	−10.5	37.6	1
3QT0	−11.2	−10.5	37.0	1
3G9E	−10.1	−9.4	36.4	1
3R5N	−10.5	−10.1	36.1	1
3NOA	−10.9	−10.3	35.4	1
6IZN	−11.2	−10.5	35.3	1
5TWO	−11.9	−11.2	35.1	1
3ADS	−9.3	−8.9	33.2	1
6ENQ	−9.9	−9.3	33.0	1
5GTN	−12.1	−11.3	32.1	1
1NYX	−11.0	−10.0	64.3	2
4R06	−10.5	−9.9	48.4	2
2HFP	−11.3	−10.3	47.1	2
3H0A	−10.8	−10.0	42.2	2
1I7I	−10.1	−9.6	40.0	2
4FGY	−10.2	−9.2	39.7	2
6MD0	−10.0	−9.5	37.5	2
6AD9	−9.9	−9.4	36.9	2
3T03	−10.2	−9.5	36.8	2
6ZLY	−10.4	−9.8	35.5	2
6AVI	−10.6	−10.0	34.8	2
6AVI	−11.0	−10.3	33.7	2
6DCU	−10.4	−9.7	31.5	2
1ZGY	−10.3	−9.5	30.4	2
2Q59	−11.3	−10.1	41.6	3
6TSG	−10.3	−9.5	30.2	4
3CWD	−9.2	−8.7	33.0	5

Supplementary Table S2a. Docking results for THC in PPARγ.

THCA				
**PDB ID**	**Lowest binding energy (Kcal/mol)**	**Mean binding energy (Kcal/mol)**	**Frequency (%)**	**Cluster**
3IA6	−10.5	−9.8	69.4	1
3ADS	−9.6	−8.9	62.6	1
6AVI	−10.7	−9.9	62.3	1
5UGM	−11.7	−10.9	58.2	1
4A4W	−9.8	−9.0	55.7	1
3G9E	−10.1	−9.3	55.1	1
1FM9	−11.1	−10.3	54.9	1
3VSO	−10.4	−9.9	48.5	1
6IZN	−11.4	−10.3	46.5	1
1I7I	−10.4	−9.5	46.4	1
6DCU	−10.2	−9.2	46.1	1
3ET3	−11.4	−10.3	43.9	1
6MD0	−10.2	−9.5	43.4	1
6JF0	−10.4	−9.5	42.6	1
4R06	−10.6	−9.8	41.5	1
3BC5	−10.9	−10.3	40.4	1
6ENQ	−10.2	−9.4	38.4	1
1NYX	−10.4	−9.6	37.8	1
6Y3U	−9.0	−8.0	37.0	1
3R8I	−10.9	−10.0	33.6	1
2ATH	−10.4	−9.8	32.5	1
1NYX	−10.1	−9.3	32.1	1
4JAZ	−11.4	−10.5	31.6	1
3VN2	−9.9	−9.0	31.1	1
4HEE	−9.5	−8.8	30.7	1
4PVU	−9.5	−8.8	30.7	1
6TSG	−10.5	−9.5	52.5	2
3TY0	−10.8	−10.0	35.2	2
5TWO	−12.1	−10.9	39.2	3
3S9S	−11.8	−10.6	34.2	3
1ZGY	−10.0	−9.1	49.4	4
3NOA	−11.0	−10.1	32.8	5

Supplementary Table S2b. Docking results for THCA in PPARγ.

CBD				
**PDB ID**	**Lowest binding energy (Kcal/mol)**	**Mean binding energy (Kcal/mol)**	**Frequency (%)**	**Cluster**
1WM0	−10.9	−9.1	29.9	1
4HEE	−10.5	−9.3	29.4	1
6DGO	−10.3	−8.9	20.0	1
6IZN	−10.3	−8.8	18.0	1
3S9S	−11.7	−9.9	17.0	1
3S9S	−10.6	−9.3	16.3	1
1NYX	−10.1	−9.0	19.8	2
2ZK6	−11.0	−9.3	17.6	2
6ZLY	−10.9	−9.0	17.6	2
6L89	−10.4	−9.0	16.6	2
3CWD	−8.7	−8.1	15.7	2
2P4Y	−9.0	−8.1	15.6	3
6DBH	−11.3	−10.1	15.5	3
6MS7	−10.6	−9.2	15.1	3
3NOA	−10.3	−9.5	17.0	4
2HFP	−11.0	−9.3	15.6	4
6D8X	−10.5	−9.2	19.3	5
2POB	−10.8	−9.5	15.6	5

Supplementary Table S2c. Docking results for CBD in PPARγ.

CBDA				
**PDB ID**	**Lowest binding energy (Kcal/mol)**	**Mean binding energy (Kcal/mol)**	**Frequency (%)**	**Cluster**
1FM9	−10.2	−8.5	17.2	1
1ZGY	−9.4	−8.2	21.8	1
2FVJ	−9.5	−7.9	17.8	1
2POB	−10.2	−8.6	15.9	1
3ADS	−9.0	−7.8	21.2	1
3G9E	−9.1	−7.8	17.6	1
3IA6	−9.3	−7.9	25.7	1
3VJH	−9.1	−7.7	15.7	1
4HEE	−8.5	−7.2	19.1	1
4PVU	−9.1	−7.7	15.6	1
6AVI	−9.5	−8.1	22.1	1
6DCU	−9.7	−8.5	20.4	1
6TSG	−8.9	−7.2	24.8	1
6Y3U	−8.1	−7.0	24.6	1
1WM0	−10.3	−8.5	26.9	2
3QT0	−9.3	−8.1	20.2	2
3R8A	−9.0	−7.6	18.3	2
3S9S	−10.7	−9.2	19.5	2
5TWO	−10.4	−8.8	23.0	2
6DBH	−10.5	−8.6	17.6	2
1NYX	−10.3	−9.3	18.5	3
1ZEO	−9.8	−8.5	21.3	3
6D8X	−9.9	−8.5	19.3	3
1WM0	−10.9	−9.2	22.0	4
3CWD	−8.7	−8.0	22.9	4
3TY0	−10.8	−9.8	15.8	4
2G0G	−10.7	−8.6	24.0	5

Supplementary Table S2d. Docking results for CBDA in PPARγ.

Receptor	Ligand	Run	Polar interaction	Aromatic-aromatic interaction	Binding Mode	Freq (%)
5UGM	THC	1	Ser342	-	1a	98
5UGM	THC	3	Ile281	-	1b	96
2HFP	THC	1	-	Tyr327	2	94
2HFP	THC	3	Leu340	-	3	84
2Q59	THC	1	Phe282, Gln286	Phe282, Phe363	4	89
2Q59	THC	2	His449	Phe363	5	92
2Q59	THC	3	Phe282, Gln286	Phe282, Phe363	4	92
6TSG	THC	1	Arg288	-	6	78
5UGM	THCA	1	Arg280, Ile281, Ser342	-	1a	99
5UGM	THCA	2	Arg280, Ser342	-	1b	94
3TY0	THCA	1	-	-	2	92
5TWO	THCA	1	Ser342	-	3	96
5TWO	THCA	2	Ser342	-	3	91
5TWO	THCA	3	Arg288, Ser342, Glu343	-	4	98
1ZGY	THCA	1	Gln286, Tyr327, Lys367	-	5a	93
1ZGY	THCA	3	-	-	6	91
3NOA	THCA	1	Arg288, Ser342	-	7	81
3NOA	THCA	2	Cys285, Ser289, Tyr327, Lys367	-	5b	98
3NOA	THCA	3	Tyr327, Lys367	-	5c	97
1WM0	CBD	3	Ser289, His449	-	1	92
2ZK6	CBD	1	-	-	2a	97
2ZK6	CBD	2	Ile281, Cys285	-	2b	86
2ZK6	CBD	3	Leu340	-	2a	98
6DBH	CBD	2	-	-	3	92
3NOA	CBD	1	Phe363	Tyr327, Phe363	4	94
6D8X	CBD	1	Cys285, Ser289, Tyr327	Tyr327	5	86
6D8X	CBD	3	Ser289, His449	-	5	93
1ZGY	CBDA	1	Asp260, Ser342	-	1a	97
1ZGY	CBDA	2	Ser342	Phe287	1a	85
1ZGY	CBDA	3	Ile267, Arg288	-	1b	97
5TWO	CBDA	2	Ser342	-	2a	98
5TWO	CBDA	3	Lys367	-	2b	93
6D8X	CBDA	1	Arg288, Ser289, Tyr327, His449	Tyr327	3	97
6D8X	CBDA	2	Cys285, Ser289, Tyr327	Tyr327	3	95
3TY0	CBDA	1	Ser342, Glu343	-	4	96
3TY0	CBDA	2	Ile281, Cys285, Arg288, Ser342	-	5	97
3TY0	CBDA	3	Ser342, Glu343	-	4	93

2G0G	CBDA	1	Phe282, Tyr327, Lys367	Phe282, Phe363	2c	94
2G0G	CBDA	2	Tyr327, Lys367	Tyr327	2c	86
2G0G	CBDA	3	Ser289, Tyr327, Lys367, His449	-	2d	93

Supplementary Table S3. Subset of MD systems selected for the analysis. For each system, the residues involved in polar and aromatic-aromatic interactions with the cannabinoid are indicated, along with the assigned binding mode and the frequency of the corresponding representative cluster.

Ligand	Receptor	Run	Mode	Resid (1)	HBO	Ene	Resid (2)	HBO	Ene	Resid (3)	HBO	Ene	Resid (4)	HBO	Ene
THC	5UGM	r1	1a	Ser342	82	−1.2 ± 0.5									
THC	2Q59	r3	4	Phe282	67	−3.1 ± 0.7	Gln286	59	−1.0 ± 0.4						
THCA	5UGM	r1	1a	Arg280	90	−9.0 ± 0.8	Ile281	66	−1.4 ± 0.2	Ser342	51	−1.0 ± 0.4			
THCA	5TWO	r3	4	Arg288	62	−9.0 ± 0.8	Ser342	97	−1.1 ± 0.4	Glu343	56	0.4 ± 0.2			
THCA	3NOA	r2	5b	Cys285	55	−2.7 ± 0.4	Ser289	66	−0.3 ± 0.2	Tyr327	89	−1.9 ± 0.6	Lys367	98	−8.8 ± 1.5
CBD	2ZK6	r2	2b	Ile281	52	−2.1 ± 1.0	Cys285	49	−2.3 ± 0.7						
CBD	6D8X	r1	5	Cys285	46	−1.8 ± 0.4	Ser289	81	−1.0 ± 0.8	Tyr327	44	−0.8 ± 0.4			
CBDA	6D8X	r1	3	Arg288	85	−8.0 ± 0.8	Ser289	67	−0.6 ± 0.5	Tyr327	76	−0.8 ± 0.5	His449	80	−0.9 ± 0.6
CBDA	3TY0	r2	5	Ile281	90	−4.0 ± 0.9	Cys285	68	−1.9 ± 0.6	Arg288	78	−7.4 ± 2.5	Ser342	51	−2.6 ± 0.9
CBDA	2G0G	r2	2c	Tyr327	99	−3.6 ± 0.7	Lys367	98	−11.3 ± 1.7						

Supplementary Table S4. Hydrogen-bond occupancies (HBO, %) and per-residue MM/GBSA decomposition energies (Ene, Kcal/mol) for residues participating in polar interactions with the ligands in the different PPARγ/cannabinoid systems.
